# Gene Association Analysis of Quantitative Trait Based on Functional Linear Regression Model with Local Sparse Estimator

**DOI:** 10.3390/genes14040834

**Published:** 2023-03-30

**Authors:** Jingyu Wang, Fujie Zhou, Cheng Li, Ning Yin, Huiming Liu, Binxian Zhuang, Qingyu Huang, Yongxian Wen

**Affiliations:** 1 College of Computer and Information Science, Fujian Agriculture and Forestry University, Fuzhou 350002, China; 2 Institute of Statistics and Application, Fujian Agriculture and Forestry University, Fuzhou 350002, China

**Keywords:** association analysis, rare variants, function linear regression model, local sparse estimation, common variants

## Abstract

Functional linear regression models have been widely used in the gene association analysis of complex traits. These models retain all the genetic information in the data and take full advantage of spatial information in genetic variation data, which leads to brilliant detection power. However, the significant association signals identified by the high-power methods are not all the real causal SNPs, because it is easy to regard noise information as significant association signals, leading to a false association. In this paper, a method based on the sparse functional data association test (SFDAT) of gene region association analysis is developed based on a functional linear regression model with local sparse estimation. The evaluation indicators CSR and DL are defined to evaluate the feasibility and performance of the proposed method with other indicators. Simulation studies show that: (1) SFDAT performs well under both linkage equilibrium and linkage disequilibrium simulation; (2) SFDAT performs successfully for gene regions (including common variants, low-frequency variants, rare variants and mix variants); (3) With power and type I error rates comparable to OLS and Smooth, SFDAT has a better ability to handle the zero regions. The *Oryza sativa* data set is analyzed by SFDAT. It is shown that SFDAT can better perform gene association analysis and eliminate the false positive of gene localization. This study showed that SFDAT can lower the interference caused by noise while maintaining high power. SFDAT provides a new method for the association analysis between gene regions and phenotypic quantitative traits.

## 1. Introduction

In recent years, with the development of high-throughput sequencing technology and the application of second-generation and third-generation sequencing platforms, unprecedented large-scale and high-dimensional genetic variation data have been generated. SNP (single nucleotide polymorphism) or CNVS (copy number variation) have become common genetic markers for studying the genetic mechanism of traits. Linkage disequilibrium-based association analysis has achieved great success in detecting the pathogenic genes of human diseases and the genetic structure of complex traits of animals and plants [1,2,3]. Genome-wide association analysis (GWAS) is a high-throughput genotyping technique, of which the millions of SNPs or CNVS use as genetic markers to identify causal genes by association analysis. GWAS has achieved primary success in the genetic studies of humans, animals and plants. GWAS falls into two categories of analysis projects: common variant association study (CVAS, Minor Allele Frequency (MAF) > 5%) and rare variant association study (RVAS, MAF <= 1~5% or MAF < 1%), where MAF <= 1~5% is called low-frequency variants and MAF < 1% is called rare variants. Most of the causal loci identified by the current GWAS study are common variants and could only explain a small proportion of the phenotypic variation. From the view of biological evolution and population genetics, most of the mutant alleles are low-frequency, and the associated loci controlling complex traits are generally low-frequency variants [4]. The association between low-frequency variation and complex traits has been reported [5,6,7], and some GWAS methods for dealing with low-frequency variants have also been proposed [8,9,10].

Univariate association detection is an effective method for common variants in gene association analysis. This analysis method has many advantages and has achieved good results through the improvement of many experts and scholars. The variants in the gene region are detected one by one while using this method. This approach can be adopted for common variants, while for rare variants, other important information contained in their genetic region may be overlooked, such as the location of individual variants, the correlation between variants, etc., so it is easy to underestimate or overestimate the power of a rare variant. To overcome this problem, many association analysis methods based on the genomic region have been proposed, including: OMNI [11], eSCAN [12], SKAT [13], SKAT-O [14], CauchyGM-O [15], tpSSU [16], etc.

There are so many association analysis methods based on genomic regions, but they could be generally divided into three kinds. The first type of method is based on merging ideas [10,17,18,19]. This type of method usually integrates all the variants into a new variable and obtains the detection power of the new variable. This is a common way is to add all variables in the region to form a new variable and then test the variable [17]. This kind of method can reduce the power’s loss due to the huge degree of freedom. The disadvantage of this method is it requires uniform direction for effect in a detection region; otherwise, it is difficult to perform an effective detection.

The second type of method is based on the variance component test of the mixed model [20,21,22,23,24]. In order to solve the directional problem of the loci effect, a variance component test was proposed. The variance component test does not focus on how to combine rare variants. It assumed the genetic effects of rare variants subject to a normal distribution. By testing the variance component of random effects, the associated relationship between rare variants and phenotypic traits could be better studied [25]. This kind of method needs to select a kernel function to measure the degree of genetic similarity between any two individuals in the same detection area. The variance component approach does not have many requirements for the effect's direction.

The third type of method is based on the functional data analysis (FDA) [26,27,28,29] proposed in recent years. Due to high-density genetic marker data, the original genetic model was transformed from a traditional multiple-linear regression model to a functional linear model (FLM). A coefficient function consisting of a set of basis functions and its coefficients could be taken from the functional linear model which represents the genetic effects. By testing coefficients in the coefficient function, we can know whether there is a significant non-zero genetic effect value in this region. Many previous studies have shown that the methods based on the functional linear regression model have higher power than those based on the merging ideas and variance component tests of the mixed model [26,27,30]. The application of the functional linear regression model in gene association analysis has been explored in many directions (additive, dominant and epistasis) [31,32], but researchers were paying more attention to the power of each method, it seems that power was the whole point of evaluating methods. Of course, the power of the method was an extremely important indicator for measuring the quality of a gene association analysis method. For this indicator, the analysis methods based on FDA performed very well, but there is still a problem: there is sparsity in the gene region, and traditional associated analysis methods do not have the capacity to shrink a sparse region, resulting in high power, but they also tend to identify some noise information as an associated signal, so if a method based on FDA could not only effectively compress the sparse region but also without reducing the power too much, then the application of this method would achieve better practical results. At the same time, if we can provide quantitative indicators to measure the impact of genetic variation on phenotypic traits and analyze the complex relationship between phenotypic traits and genetic loci, it will be more accurate to analyze the impact of genetic variation on phenotypic traits.

Lin et al. [33] proposed the fSCAD (functional smoothly clipped absolute deviation) method, which improved the original FLM by adding a SCAD (smoothly clipped absolute deviation) penalty item based on FLM. This method can accurately compress the zero areas of the model region to zero without excessively compressing the non-zero area of the model region, which also means that this method can allow unassociated variants to be ignored in gene association analysis and remain the true associated variants.

In consideration of these advantages of fSCAD and the problems of gene association analysis methods based on FDA, a new gene association analysis approach called sparse functional data association test (SFDAT) which is based on FDA [33] was proposed in this paper, and the computer simulation was used to evaluate the effect coefficient estimation accuracy, the type I error rate and power. The real data set of *O. sativa* was analyzed by SFDAT to demonstrate the applicability of real data of SFDAT.

## 2. Theory and Methods

### 2.1. Genetic Model

Let $y_{i}$ be the phenotypic value of *i*th individual. For *i-*th individual, the traditional linear genetic model can be expressed as:

(1) $$\begin{array}{c} y_{i}=\mu +\sum_{j=1}^{K}x_{ij}\beta_{j}+\varepsilon_{i}\;\;\;\;i=1,2,\cdots ,n \end{array}$$

where $x_{ij}$ is a genotype profile (if A and a represent a pair of alleles, then when the genotype is AA, $x_{ij}$ is taken as 2; when the genotype is Aa, it is taken as 1; when the genotype is aa, it is 0). $\beta_{j}$ represents the effect coefficient of genetic marker, $\varepsilon_{i}∼N\left(0,\;\sigma^{2}\right)$, $\sigma^{2}$ is the environmental genetic variance, K is the number of genetic markers. With the increase in the number of genetic markers, the degree of freedom gradually increases, and the multi-collinearity among variables becomes more and more serious, eventually leading to the reduction in estimation accuracy and power. This is especially true when the genetic markers are low-frequency variations. In order to reduce the degree freedom of the model and the multiple collinearities of variables due to low-frequency variation, the functional linear model (FLM) can be used instead of the multiple linear genetic model:

(2) $$\begin{array}{c} y_{i}=\mu +\int_{0}^{T}X_{i}\left(t\right)\beta \left(t\right)dt+\varepsilon_{i}\;\;\;i=1,2,\cdots ,n \end{array}$$

where $\varepsilon_{i}$ is an independent and normal distribution with zero mean and variance $\sigma^{2}$, [0, *T*] represents the genomic region under consideration, that is, a DNA fragment that contains multiple SNP loci, among which there may be SNP loci that can affect the target quantitative trait. The discrete genetic markers in Equation (1) are converted into the continuous genetic marker function, and the effects of genetic markers $\beta_{j}$ are also converted into a continuous genetic effect function β(t).

For Equation (2), the B-spline function is used to fit the genetic variants and the genetic effects. According to the functional data analysis method [26,34]: First, let l variants be in a sequence of their physical locations $t_{0}<t_{1}\leq t_{2}\cdots \leq t_{l}<t_{l+1}<\cdots <t_{T}=T$ which constitutes the genomic region [0,T]; Second, a series of B-spline basis functions are defined and let $B_{k}\left(t\right)$ be a B-spline basis function; Third, define M+1 equidistant nodes $0=v_{0}<v_{1}<\cdots <v_{M}=T$ in the interval [0,T]. After that, these discrete genetic variants can be expanded as a continuous function:

(3) $$\begin{array}{c} X_{i}\left(t\right)=\sum_{k=1}^{K_{G}}u_{ik}B_{k}\left(t\right) \end{array}$$

where the coefficient could be obtained by minimizing the following equation:

(4) $$\begin{array}{c} \sum_{j=1}^{T}[X_{i}\left(t_{j}\right)-\sum_{k=1}^{K_{G}}B_{k}\left(t_{j}\right)u_{ik}{]}^{2} \end{array}$$

Let $X_{i}={\left[X_{i}(t_{1}),\cdots ,X_{i}(t_{T})\right]}^{T}$, $u_{i}={\left[u_{i1},\cdots u_{iK_{G}}\right]}^{T}$, $B=\left[\begin{array}{ccc} B_{1}(t_{1}) & \cdots & B_{K_{G}}(t_{1})\\ \cdots & \cdots & \cdots \\ B_{1}(t_{T}) & \cdots & B_{K_{G}}(t_{T}) \end{array}\right]$.

The coefficient $u_{ik}$ is estimated to be ${\hat{u}}_{i}={\left(B^{T}B\right)}^{-1}B^{T}X_{i}$. Similar to the genetic variants, the genetic effects also can be expanded as

(5) $$\begin{array}{c} \beta \left(t\right)=\sum_{k=1}^{K_{\beta }}\beta_{k}B_{k}\left(t\right)=\theta^{T}\left(t\right)\beta \end{array}$$

where $\theta (t)={\left[B_{1}(t),\cdots ,B_{K_{\beta }}(t)\right]}^{T}={\left[\theta_{1}(t),\cdots ,\theta_{K_{\beta }}(t)\right]}^{T}$, $\beta ={\left[\beta_{1},\cdots ,\beta_{K_{\beta }}\right]}^{T}$.

Let $Y={\left[y_{1},\cdots ,y_{n}\right]}^{T},X(t)={\left[X_{1}(t),\cdots ,X_{n}(t)\right]}^{T},U={\left[u_{1},\cdots u_{n}\right]}^{T},B(t)={\left[B_{1}(t),\cdots ,B_{K_{G}}(t)\right]}^{T}$,$I={\left(1,1,\cdots ,1\right)}^{T}$, $\varepsilon ={\left[\varepsilon_{1},\varepsilon_{2},\cdots ,\varepsilon_{n}\right]}^{T}$, then X(t)=UB(t).

The functional linear model of Equation (2) can be rewritten as

(6) $$\begin{array}{c} \begin{array}{c} Y=\mu I+\int_{0}^{T}UB\left(t\right)\theta^{T}\left(t\right)\beta dt+\varepsilon \\ \;\;\;\;\;\;\;\;\;=\mu I+U\left[\int_{0}^{T}B\left(t\right)\theta^{T}\left(t\right)dt\right]\beta +\varepsilon \end{array} \end{array}$$

Let $J_{B\theta }=\left[\begin{array}{ccc} \int_{0}^{T}B_{1}(t)\theta_{1}(t)dt_{} & \cdots & \int_{0}^{T}B_{1}(t)\theta_{K_{\beta }}(t)dt\\ \cdots & \cdots & \cdots \\ \int_{0}^{T}B_{K_{G}}(t)\theta_{1}(t)dt & \cdots & \int_{0}^{T}B_{K_{G}}(t)\theta_{K_{\beta }}(t)dt \end{array}\right]$ $W=UJ_{B\theta }$, then Equation (6) can be rewritten as

(7) $$\begin{array}{c} Y=\mu I+W\beta +\varepsilon \end{array}$$

The form of the linear regression equation is:

(8) $$\begin{array}{c} y=\mu +w_{1}\beta_{1}+w_{2}\beta_{2}+\cdots +w_{K_{\beta }}\beta_{K_{\beta }}+\varepsilon =\mu +\sum_{i=1}^{K_{\beta }}w_{i}\beta_{i}+\varepsilon \end{array}$$

In the actual operation, the integral interval [0, T] can be converted into [0, 1].

### 2.2. Parameter Estimation

It can be seen from the equations established above that the genetic variants and effects in the functional linear genetic model are transformed into a continuous function through B-spline. Finally, the functional genetic model is transformed into the traditional linear regression model. In order to obtain the local sparse estimation of β(t), we use the method of parameter estimation based on the penalty function proposed by Lin et al. [33]. Lin et al. [33] proposed that $\hat{\beta }\left(t\right)$ and $\hat{\mu }$ in Equation (2) could be estimated by minimizing the following loss function:

(9) $$\begin{array}{ll} Loss\left(\beta ,\;\mu \right)= & \frac{1}{n}\sum_{i=1}^{n}[y_{i}-\mu -\int_{0}^{T}X_{i}\left(t\right)\beta \left(t\right)dt{]}^{2}+\gamma ∥D^{m}\beta {∥}^{2}\\ & +\frac{M}{T}\int_{0}^{T}p_{\lambda }\left(\left|\beta \left(t\right)\right|\right)dt \end{array}$$

where d,M,T as defined above, $D^{m}$ is the m order differential operator, m≤d, which we usually take m=2, ‖•‖ is $L^{2}$ norm. As defined by Fan and Li’s [35] $p_{\lambda }\left(•\right)$ is:

(10) $$\begin{array}{c} p_{\lambda }\left(u\right)=\{\begin{array}{l} \lambda u\;\;0\leq u\leq \lambda \\ -\frac{u^{2}-2a\lambda u+\lambda^{2}}{2\left(a-1\right)}\;\;\lambda <u<a\lambda \\ \frac{\left(a+1\right)\lambda^{2}}{2}\;\;\;\;\;u\geq a\lambda \end{array} \end{array}$$

Its domain is [0, +∞], the value of a could be 3.7 which is suggested by Fan and Li [35] and the value of λ is determined by the sample size. The $\gamma {\left\|D^{m}\beta \right\|}^{2}$ term in the loss function Loss(β,μ ) is the roughness penalty of β(t), it controls the smoothness of β(t), where parameter γ can further adjust the severity of roughness penalty, γ is called the smoothing parameter. Due to the roughness penalty, the functional linear regression model FLM has a certain ability to resist “noise”. $\frac{M}{T}\int_{0}^{T}p_{\lambda }(\left|\beta (t)\right|)dt$ is the local sparse penalty of β(t), it can compress the tiny β(t) directly to zero. The parameter λ determines how tiny value would be compressed, λ is called the compression parameter. In addition, the local sparse penalty will play different constraints according to the specific form of β(t).

For Equation (9), when γ=0, λ=0, Loss(β,μ ) is the same as the loss function of ordinary functional linear regression model (FLM), the method of parameter estimation is called Ordinary Least-Square Estimator (OLS); when γ≠0,λ=0, Loss(β,μ ) equals to the loss function of the smoothed functional linear regression model, the method of parameter estimation is called Smoothing Spline Estimator (Smooth); when γ≠0, λ≠0, Loss(β,μ ) is a loss function for the functional linear regression model with locally sparse. The method of parameter estimation is called smooth and locally sparse (SLoS) estimator. There are two advantages of SLoS’s loss function: first, these rough results due to false correlation effects could be smoothed by the roughness penalty; second, the small and insignificant effects would be directly compressed to zero, which further reduces the false positive.

### 2.3. Test Statistics

Another major problem in the genetic study for quantitative traits is whether the association between genetic regions and phenotypic traits is real existence. In general, we consider the following hypothesis-testing questions:

$$H_{0}:\beta \left(t\right)=0\;,\;H_{1}:\;\beta \left(t\right)\neq 0,\;\mathrm{for}\;\mathrm{any}\;t\in \left[0,\;T\right]$$

Since the genetic effect function is the expansion of the basis function, the above assumption is equal to the following assumptions:

$$H_{0}:\mathrm{for}\;\mathrm{any}\;\beta_{i}=0,i=1,2,\cdots ,K_{\beta }.,\;H_{1}:\beta_{i}\;\mathrm{not}\;\mathrm{all}\;\mathrm{zero},\;i=1,2,\cdots ,K_{\beta }.$$

For

(11) $$\begin{array}{c} y=\mu +\sum_{i=1}^{K_{\beta }}w_{i}\beta_{i} \end{array}$$

The statistic can be defined as:

(12) $$\begin{array}{c} F=\frac{\frac{RSS}{K_{\beta }}}{\frac{ESS}{n-K_{\beta }-1}}∼F\left(K_{\beta },\;n-K_{\beta }-1\right) \end{array}$$

where RSS is the regression square sum of Equation (11), and ESS is the residual square sum of Equation (11).

The above statistical test is for the entire gene region [0, T], let us move on to the test for the subgene area. Suppose N(β) is the zero value area of β(t) and S(β) is non-zero value area of β(t), then N(β)={t∈[0,T]:β(t)=0}, S(β)={t∈[0,T]:β(t)≠0}. The estimation $\hat{\beta }(t)$ has Oracle property for N(β) by SLoS estimator. Lin [33] have proven the following conclusion: if β(t)=0 for any t∈[0,T], then the estimation of $\hat{\beta }(t)=0$ for any t∈[0,T] in probability. In other words, if the estimation of $\hat{\beta }(t)\neq 0$ for any t∈[0,T], then β(t)≠0 for any t∈[0,T] in probability. Suppose $N(\hat{\beta })$ is the zero value area of $\hat{\beta }(t)$ and $S(\hat{\beta })$ is the non-zero value area of $\hat{\beta }(t)$, $N(\hat{\beta })$ is convergence to N(β) in probability and $S(\hat{\beta })$ is convergence to S(β) in probability. Therefore, the zero and non-zero area of $\hat{\beta }(t)$ represent that of β(t). The view of statistical genetic, the effect function $\hat{\beta }(t)$ to be zero means that there is no correlation between phenotypic traits and locus t; the effect function $\hat{\beta }(t)$ to be non-zero, it means that there is a correlation between phenotypic traits and locus t.

Therefore, it is necessary to establish indicators for SFDAT to estimate the effect function in zero or non-zero regions. On the one hand, it can measure the accuracy of the model estimation; on the other hand, it can provide a reference for a more accurate analysis of functional linear model regional association.

### 2.4. Indicators of Estimated Accuracy

There are numerous SNP loci in the gene region, and the identified causal SNP loci will inevitably have location deviation, so we regard the region with a total of 200 loci centered on the causal SNP loci as the region of acceptable deviation (Abbr. RAD), that is, we can accept that the identified causal SNP is within the RAD. Then, on the basis of being closer to reality, in order to evaluate the ability of the function to compress the zero regions on the one hand, and measure the accuracy of the function to identify the non-zero region on the other hand, we evaluate the identification ability and the region-selection ability of the model through the correct selection ratio (Abbr.CSR) of zero regions outside RAD and the discovery length (Abbr. DL) for non-zero regions in RAD, which was defined, respectively, by

(13) $$\begin{array}{c} CSR=\frac{S_{0}\left(\hat{\beta }\left(t\right)\cap^{\;}\beta \left(t\right)\right)}{S_{0}\left(\beta \left(t\right)\right)} \end{array}$$

(14) $$\begin{array}{c} DL=S_{1}\left(\hat{\beta }\left(t\right)\right) \end{array}$$

$S_{0}\left(\beta \left(t\right)\right)$ and $S_{1}\left(\beta \left(t\right)\right)$ are denoted as the length of β(t) in zero region and non-zero region. β(t) and $\hat{\beta }\left(t\right)$ represent the real and estimated effect values at the locus t, respectively. For a good test method, its CSR should be enough large to handle non-association signals region effectively; in the meantime, the more precise ability to identify the association signals, the lower DL it has.

For areas with zero or non-zero effects in the integral region, the following integral squared errors (ISE) are defined by Lin [33]:

(15) $$\begin{array}{c} ISE_{0}=\frac{1}{l_{0}}\int_{N\left(\beta \right)}{\left(\hat{\beta }\left(t\right)-\beta \left(t\right)\right)}^{2}dt\;and\;ISE_{1}=\frac{1}{l_{1}}\int_{S\left(\beta \right)}{\left(\hat{\beta }\left(t\right)-\beta \left(t\right)\right)}^{2}dt \end{array}$$

where $l_{0}$ is the length of zero areas, $l_{1}$ is the length of non-zero areas. ISE_0_ and ISE_1_ can be used to estimate the error between estimated $\hat{\beta }(t)$ and true β(t) on zero and non-zero areas, respectively. In addition to the performance of model prediction, it is judged by prediction mean squared errors (PMSE):

(16) $$\begin{array}{c} PMSE=\frac{1}{N}\sum_{\left(x,y\right)\in test}(y-\hat{\mu }-\int_{0}^{T}X\left(t\right)\hat{\beta }\left(t\right)dt{)}^{2} \end{array}$$

where *test* is the test individual set, N is the number of samples, $\hat{\mu }$ and $\hat{\beta }\left(t\right)$ are estimated of μ and β(t).

According to the above definition, we can define ISE_0_, ISE_1_ and PMSE as criteria for evaluating the accuracy of effect estimates for gene regions. To determine the degree of fitting on zero effects, we define

(17) $${\mathrm{ISE}}_{0}=\frac{1}{|A_{0}|-1}\sum_{t\in A_{0}}{\left(\hat{\beta }\left(t\right)-\beta \left(t\right)\right)}^{2}$$

$A_{0}$ denotes a set of variants loci in which no association exists, $|A_{0}|$ denotes the number of elements in set $A_{0}$. $\hat{\beta }\left(t\right)$ and β(t), respectively, denote estimated effects and actual effect values on locus t in set $A_{0}$. It indicates the degree of the overall deviation of the true and estimated values at the zero effects, the lower ISE_0_, the more accurate estimation of zero effects. To determine the degree of fitting on the non-zero effects, we define

(18) $${\mathrm{ISE}}_{1}=\frac{1}{|A_{1}|-1}\sum_{t\in A_{1}}{\left(\hat{\beta }\left(t\right)-\beta \left(t\right)\right)}^{2}$$

$A_{1}$ represents the set of associated variants loci in the region, $|A_{1}|$ represents the number of elements in set $A_{1}A_{1}$. $\hat{\beta }\left(t\right)$ and β(t) represent the estimated and actual effect values on locus test in set $A_{1}$, respectively. It indicates the degree of overall deviation between true and estimated values at the non-zero effects, the lower ISE_1_, the more precise estimation of non-zero effects. To determine the degree of fit for the genetic model, we define

(19) $$PMSE=\frac{1}{N-1}\sum_{y_{i}\in test}{\left(y_{i}-{\hat{y}}_{i}\right)}^{2}$$

test represents the test individual set, N represents the number of individuals in the test set, $y_{i}$ represents the true effect value of ith individual in the test set and ${\hat{y}}_{i}$ represents the predicted value of ith individual in the test set, which indicates the overall deviation degree between true and estimated trait values at the test set, the lower PMSE, the more powerful predict ability.

### 2.5. Determine Tune Parameters

In the SFDAT method, the choice of parameter M value is not very important [36] as long as the selected M is large enough to reflect the local appearance of β(t) (including the area of zero). For selection of γ and λ, a series of candidate values are given and find out the optimal parameters based on cross-validation, generalized cross-validation, BIC (Bayesian information criterion), AIC (Akaike information criterion) or RIC (Risk Inflation Criterion).

## 3. Simulation Studies

In order to verify the feasibility and effectiveness of the SFDAT method, a computer simulation was carried out. The simulated SNP genotype data were used to study the power, type I error rate and estimation accuracy of the method. To compare the advantages of the SFDAT method, the OLS and Smooth methods were also adopted. The computer simulation code was written in R language.

The power of the model and type I error rate can be obtained by the following steps: firstly, test Equation (2) to obtain the *p* value of the genetic model under different assumptions; secondly, count the number of *p* values less than a certain threshold; thirdly, the counted number divided by the total number of simulations, and then the ratio under the non-zero hypothesis is the power and the ratio under the null hypothesis is the type I error rate. The reason for calculating in this way is: under a non-zero hypothesis, there is an associated variant in the region, if the *p* value is less than threshold α at this time, the phenotype trait and gene region have an associated relationship, so the ratio is the power; under the null hypothesis, there is no associated variant in the region, if the *p* value is still less than threshold α at this time, the phenotype trait and gene region have a false associated relationship, so this ratio become the type I error rate.

At last, a test set for each simulation (an additional 100 individuals from the simulated SNP genotype data) was generated to calculate the PMSE.

### 3.1. Simulated SNP Genotype Data

In the simulation, we consider the linkage equilibrium and linkage disequilibrium simulation. For the linkage equilibrium simulation, the simulated SNP genotype dataset consists of many simulated gene regions, each of which contains 900 SNPs, and the MAF of SNPs within each region is generated by uniform distribution U(a,b). In fact, we generate the MAF of each SNP through uniform distribution, then generate the corresponding SNP through the MAF, and finally, form the simulated gene region from these SNPs. For the generation of the simulated SNP genotype of linkage disequilibrium simulation, we refer to Wang and Pan [37,38] and set the measure of linkage disequilibrium 0.2 in the simulation.

In the simulation, the number of basis functions $K_{G},K_{B}$ is 15, the order d is 4, the node M is 11. A set of smoothing parameters γ [$10^{2}$,$10^{3}$,$10^{4}$,$10^{5}$,$10^{6}$] and a set of compression parameters λ [0.03, 0.04, 0.05, 0.06] are given here. In the calculation process, the optimal parameters will be automatically selected according to BIC.

For linkage equilibrium and linkage disequilibrium simulation, four kinds of gene regions will be discussed: rare variants gene regions, low-frequency variants gene regions, common variants gene regions and mixed variants gene regions. The rare variant's gene region is constituted by rare variants of which MAFs are generated by uniform distribution U(0.0005,0.01); The low-frequency variants gene region is constituted by low-frequency variants of which MAFs are generated by uniform distribution U(0.01,0.05); The common variants gene region is constituted by common variants of which MAFs are generated by uniform distribution U(0.05,0.5); The mixed variants gene region is randomly composed by 60% rare variants, 15% low-frequency variants, and 25% common variants.

Simulated phenotypic trait values were generated by $y=\mu +\sum_{i\in A}x_{i}\beta_{i}+\varepsilon$, where A is the set of causal SNPs, μ=1 , ε∼N(0,1). Two different types of simulation cases were considered:

Case I: A total of three scenarios will be considered: Scenario I: setting a positive causal SNP at locus 450 in the gene region; Scenario II: setting a positive causal SNP at locus 100 and 800 in the gene region, respectively; Scenario III: setting a positive causal SNP and a negative causal SNP at locus 100 and 800 in the gene region, respectively. The effect size of each scenario is fixed at 5 (β = 5).

Case II: The value of genetic effect β is $\ln(c)\times |\log_{10}(\mathrm{MAF})|/2$ (MAF is the minor allele frequency of the SNP). A total of 27 scenarios will be considered: the number of causal SNPs is 5, 10 or 20. Namely, the associated variants proportion of gene region (900 SNP) is 1/180, 1/90 and 1/45 ; the proportion of negative effect in causal SNPs was 0%, 20%, or 40%; The parameter c in the genetic effect $\ln(c)\times |\log_{10}({\mathrm{MAF}}_{i})|/2$ equals to 3, 5 or 7.

**Figure 1:**
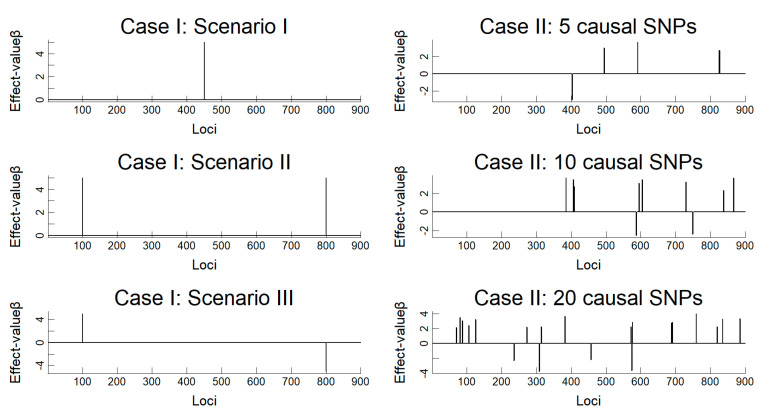
The simulated gene regions of Case I and Case II. X-axis (Loci) represents the SNP loci in the gene region, Y-axis (Effect-value β) represents the effective value of each SNP loci. The proportion of negative effect in causal SNPs and the effect size c in effect model is 20% and 3, respectively.

The simulated gene regions of Case I and Case II were shown in Figure 1. For each case, we generated 2000 samples for each gene region to simulate, and all simulations were replicated 1000 times. CSR, DL will be calculated in Case I and power, ISE_0_, ISE_1_ and PMSE will be calculated in Case II.

### 3.2. The Power Evaluation and the Estimation of Indicators

**Figure 2:**
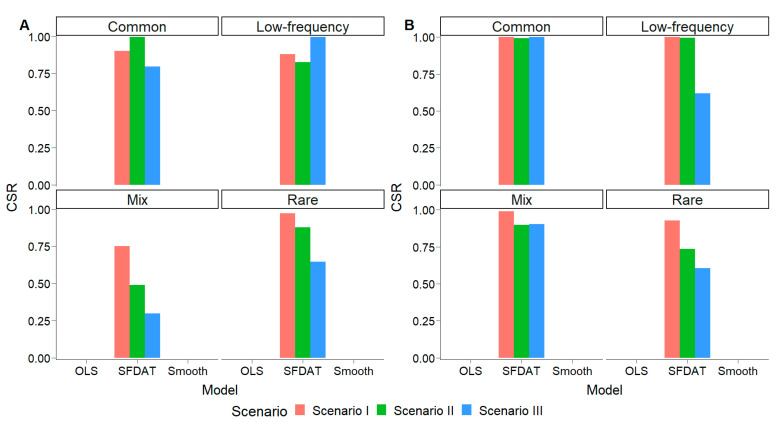
The simulation CSR of four variants using OLS, SFDAT and Smooth under three scenarios of Case I. (**A**): The situation of linkage equilibrium; (**B**): the situation of linkage disequilibrium; Scenario I: Set a positive causal SNP at locus 450 in the gene region. Scenario II: Set a positive causal SNP at locus 100 and 800 in the gene region, respectively. Scenario III: Set a positive causal SNP and a negative causal SNP at locus 100 and 800 in the gene region, respectively.

**Figure 3:**
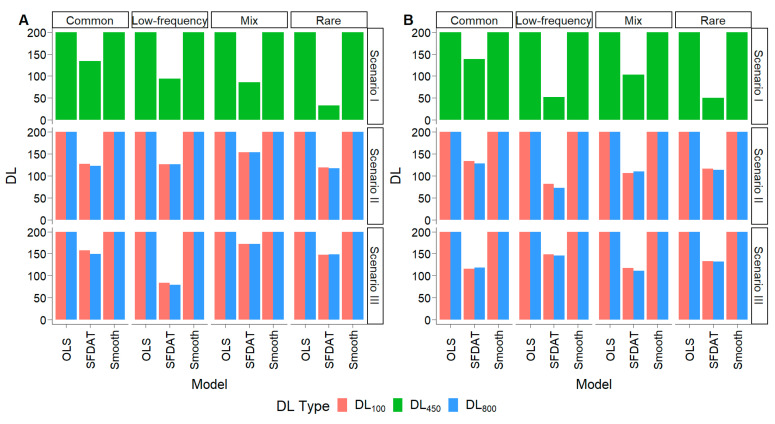
The simulation DL of four variants using OLS, SFDAT and Smooth under three scenarios of Case I. DL_100_, DL_450_, DL_800_ denotes the discovery length for non-zero region at locus 100, 450 and 800, respectively. (**A**): The situation of linkage equilibrium; (**B**): the situation of linkage disequilibrium; Scenario I: Set a positive causal SNP at locus 450 in the gene region. Scenario II: Set a positive causal SNP at locus 100 and 800 in the gene region, respectively. Scenario III: Set a positive causal SNP and a negative causal SNP at locus 100 and 800 in the gene region, respectively.

Figure 2 and Figure 3 show the CSR and DL of OLS, Smooth, and SFDAT in the three scenarios of Case I, respectively. Note that SFDAT can compress β(t) to 0 in the region of most unassociated SNPs. However, neither OLS nor Smooth has this ability, which results in their failure to compress unassociated SNP loci, that is, OLS and Smooth estimate the coefficients of these SNP loci with no genetic effect as non-zero. The ability of SFDAT to compress the non-effect region in common variants and low-frequency variants is stronger than that of mixed variants and rare variants, indicating that the gene regions with rare variants may limit the compression ability of SFDAT. SFDAT performs better in gene regions with only one causal SNP, and worst in gene regions with a positive and negative causal SNP. Meanwhile, compared with linkage equilibrium, SFDAT performs better in the simulation of linkage disequilibrium. As can be seen from Figure 3, OLS, Smooth and SFDAT can all find causal signals in RAD, but the signal regions found by SFDAT are more concentrated. OLS and Smooth explore the causal signal at each locus in the RAD, while SFDAT only identified the causal SNPs in part of the RAD under all cases. This is because OLS and Smooth do not have the ability to compress the zero region, resulting the noise fluctuations when estimating the effect functions on the unassociated SNPs loci, which mistakenly deems all the loci have the effect. SFDAT cannot only perfectly detect unassociated SNPs regions, but also accurately identify causal SNP loci. When there is only one causal SNP in the gene region, SFDAT can detect the locations of rare variants more accurately. The DL_100_ probed by SFDAT is similar to DL_800_ under scenario II and scenario III (Gene regions exist two causal SNPs). In general, the DL of linkage disequilibrium simulation is smaller than that of linkage equilibrium, that is, the location of the identified causal SNPs is more concentrated.

Table 1 illustrates the power of three methods of Case II under the significant level 0.01. As can be seen from Table 1, under the simulation of linkage equilibrium, the power for common variant regions and low-frequency variant regions are similar, the powers of rare variants regions and mixed variants regions are similar. The powers of the former (common variants and low-frequency variants) are higher than that of the latter (rare variants and mixed variants), and the powers of rare variant regions are also lower than that of mixed variant regions. That is because the former does not contain rare variants, the latter contains rare variants and the rare variants contained in the mixed variants regions will be fewer than the rare variants regions, indicating that the gene regions that do not contain rare variants have a higher power, the power of the gene region containing common variants is higher than that of a gene region containing only rare variants. For common variant regions and low-frequency variant regions, there are similar powers in various situations for the OLS, Smooth and SFDAT methods, and the OLS method is slightly better. However, for rare variants regions and mixed variants regions, the power of the OLS method is significantly better. Obviously, the powers of methods are higher when the number of causal SNPs or the effect size increases, but when the proportion of negative effect increases, the powers of methods are just the opposite. In rare variant regions, the detection results are unstable while the causal SNPs contained in the gene region become less. Finally, it has a higher power in all cases, when the number of pathogenic SNPs in the gene region reaches 20. It is shown that similar performance patterns are observed in linkage disequilibrium. However, compared with the simulation of linkage equilibrium, the power of linkage disequilibrium has improved enormously. This is owing to the overall effect of gene regions as the correlation of loci.

The ISE_0_ and its standard deviation of three methods for linkage equilibrium and linkage disequilibrium under Case II are shown in Table 2. From Table 2, under the simulation of linkage equilibrium, the standard deviation increases with the MAF decreases, it shows that the common variants fit better in the region where the effect is zero; the ISE_0_ value or standard deviation of the OLS method is the largest among three methods, while there are smaller and similar results for the Smooth and SFDAT methods; the ISE_0_ standard deviation of the Smooth method has a larger deviation than that of SFDAT method when the number of causal SNPs in the gene region is small; the ISE_0_ values are similar (in other words the degree of the fitting is close) when the number of the causal SNPs and the effect size are the same regardless of the proportion of the negative effect; the ISE_0_ and its standard deviation increase while the number of the causal SNPs and the effect size increase. Compared to the simulation of linkage equilibrium, ISE_0_ decreased significantly in the regions of common variants and mixed variants under linkage disequilibrium, and slightly increased in the regions of low-frequency variants and rare variants. It is also verified that the models can better fit the zero region of common variants.

Table 3 displays the ISE_1_ and its standard deviation of three methods under linkage equilibrium and linkage disequilibrium based on Case II. In the situation of linkage equilibrium, the standard deviation increases as MAF decreases which indicated that three methods fitted better on the common variants in the non-zero region; the ISE_1_ and its standard deviation of the three methods were similar: as the effect size c or the proportion of the negative effects increases. Under linkage disequilibrium, the results of OLS, Smooth and SFDAT in fitting non-zero regions of common variants, low-frequency variants and rare variants decreased slightly. When fitting the rare variants that do not exist negative causal SNPs, the fitting results are also slightly lower than that of linkage equilibrium, but the ISE_1_ of linkage disequilibrium simulation is significantly lower than that of linkage equilibrium when the rare variants region exists negative causal SNPs.

PMSE and its standard deviation of three methods under linkage equilibrium and linkage disequilibrium in Case II are shown in Table 4. In general, the functional-based genetic model containing rare variants fits better than those containing common variants. PMSE and its standard deviation of the three methods are similar. PMSE and its standard deviation increase with the number of causal SNPs or effect size, while the proportion of negative effect only has little influence on it. Compared with linkage equilibrium simulations, PMSE and its standard deviation of linkage disequilibrium decrease significantly in common variants, low-frequency variants and rare variants, but slightly increase in mixed variants.

### 3.3. The Type I Error Rate

We set five sample sizes of 500, 1000, 1500, 2000 and 2500, each sample size is simulated 10,000 times. Simulated traits are generated by model y=μ+ε, where μ=1, ε∼N(0,1). The significance levels are 0.05, 0.01, 0.001 and 0.0001. Table 5 summarizes the type I error rates of OLS, Smooth and SFDAT under the linkage equilibrium and disequilibrium simulation. It can be seen from Table 5 that Smooth and SFDAT controlled type I error rates correctly across all sample sizes and all significance levels. While the type I error rates of OLS is severely inflated, which means the use of OLS for gene association analysis produces false positives that can lead to false associations. Note that the SFDAT method will appear conservative when the sample size and significance level are relatively small, but as the two increase, the SFDAT method also reaches a sufficient level of significance. Relative to linkage equilibrium, the type I error rates of the three models under linkage disequilibrium increase in smaller sample sizes (500, 1000, 1500) and decrease in larger sample sizes (2000, 2500).

Combining Figure 2 and Figure 3 and Table 1, Table 2, Table 3, Table 4 and Table 5, SFDAT has a competitive performance to the OLS and Smooth in terms of location of non-causal SNP regions and identification of causal SNPs regions, as well as power, the type I error rates and other indicators. It is appreciable that OLS has relatively higher power, but its ISE_0_, the standard deviation of ISE_0_ and type I error rates are larger than those of Smooth and SFDAT methods, which declares that there may be a false correlation in the association analysis using OSL method, and the zero effect may be recognized as a non-zero effect. In addition, although Smooth has a similar performance to SFDAT in power, ISE_1_ and PMSE, it does not have the capacity to shrink sparse regions, which leads to noise fluctuations in the application of real data usually and further causes false association. Therefore, if we consider the SFDAT’s performance of the power, type I error rates and combine its performance of other indicators (the CSR, DL, ISE_0_, ISE_1_ and PMSE), the SFDAT method would be an excellent method which has extraordinarily high power and has a marvelous ability to reduce false positives.

## 4. Application to *O. sativa* Data Set

We apply SFDAT to the data set of 413 diverse rice (*O. sativa*) varieties from 82 countries [39] to demonstrate the applicability of SFDAT based on the above simulation. This data set broadly includes six categories of phenotype: plant morphology-related traits; yield-related traits; seed and grain morphology-related traits; stress-related phenotypes; cooking, eating and nutritional-quality-related traits; and plant-development-related traits. Almost all phenotypes were measured in the field at Stuttgart, Arkansas, the measuring times were repeated two times each year during the growing season (May–October) in 2006 and 2007. For details of experimental design, see Zhao [39].

Zhao [39] verified two candidate genes related to Culm habit, *D10* and *D14*. So, Culm habit and its causal SNP *D10* and *D14* are chosen for the association analysis test for displaying the feasibility of SFDAT in the real application. The samples with missing values were eliminated and matched with the genotype data, the remaining 356 samples were for follow-up analysis. The genotype data contains a total of 44,100 markers on 12 chromosomes. The missing genotype was estimated, and SNPs with a minimum allele frequency of less than 0.005 were deleted. Finally, there were 32,185 SNPs left. Each chromosome is sequentially divided into several gene regions which contains 1000 SNPs, and merges the set of less than 1000 SNPs in the chromosome with the previous gene region, 24 gene regions are obtained finally. Causal SNP *D10* and *D14* are located in the 4th and 9th gene region, respectively. The number of SNPs and the *p*-value of the association analysis of each gene region are shown in Table 6. Significant SNP loci have been identified in the 3rd, 4th, 9th and 14th gene regions, which means that SFDAT can detect the gene regions where causal SNP is located precisely. The calculation of the whole process was taken 37 s on the Intel Core 2.50 GHz CPU. This result indicates that the genetic region association analysis method based on the SFDAT method is virtually and computationally feasible.

**Figure 4:**
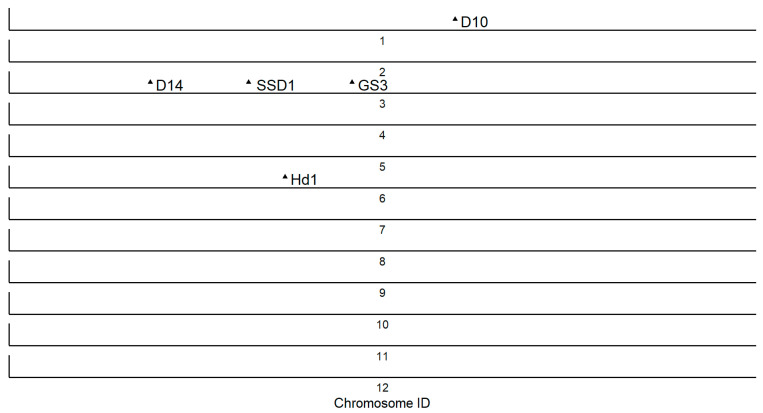
The position of SNPs that in *O. sativa* data analysis on chromosomes. Chromosome ID is the numbering of each chromosome. *D10* and *D14* are the causal SNPs of culm habit; *SSD1*, *GS3* and *Hd1* are the candidate SNPs of florets per panicle, brown rice seed length and flowering time at Aberdeen, respectively.

Then, in order to compare the capability of test in real data application of OLS, Smooth and SFDAT, the florets per panicle, brown rice seed length and flowering time in Aberdeen are chosen to be the phenotype for subsequent analysis, *SSD1*, *GS3* and *Hd1* are chosen, respectively, for the candidate SNP of these phenotypes, which had been verified by Zhao, and the locations on chromosomes of these candidate SNPs were shown in Figure 4. The phenotypic and genotype data are taken from the same process as above, each phenotype left 383, 350 and 334 samples for follow-up analysis. We regard a set of 1000 SNPs as a gene region to be tested. For each candidate SNP, we divide its surrounding region containing a total of 8000 markers into eight gene regions to be tested and ensure that these gene regions have no significant SNP associated with phenotype except the candidate SNP. *SSD1*, *GS3* and *Hd1* are, respectively located in the 6th, 4th and 3rd gene regions of the eight gene regions to be ested. We perform association analysis on the eight gene regions to be detected for each candidate SNP. The associated gene regions detected by OLS, Smooth and SFDAT at different significant levels are shown in Table 7. OLS, Smooth and SFDAT can detect the gene region of candidate SNP at different significant levels. However, severe false correlations appear in OLS and Smooth. OLS and Smooth detect significant SNP loci in the gene regions without candidate SNP, especially in the association analysis of the eight gene regions of *GS3*, OLS and Smooth consider that all gene regions contained significant SNP loci when α is equal to 0.05, 0.01 and 0.001. SFDAT show a robust ability for accurate positioning. Compare with OLS and Smooth, SFDAT can shrink the regions without candidate SNP and accurately identify the regions containing candidate SNP. SFDAT detects some gene regions that do not contain candidate SNPS at some level of significance, but these gene regions are close to the gene regions where SNP candidates were located.

## 5. Discussion

GWAS is a new strategy that uses millions of SNPs in the genome as molecular genetic markers to conduct genome-wide comparative analysis or association analysis, and to find out the genetic variation that affects complex traits through comparison. In 2005, *Science* magazine reported the first age-related GWAS study of macular degeneration [40]. For more than a decade, research on genome-wide association analysis has grown rapidly, but most methods are aimed at common variants. In recent years, more and more scholars have begun to pay attention to the study of rare variants. With the development of a new generation of high-throughput sequencing technology, TB or even more sequence data will be generated every day, and the data will be gradually changed from discrete to dense. We can regard it as continuous data, and thus functional data analysis methods have emerged. It can be seen from the above analysis that it can analyze both common and rare variants [26]. In recent years, more and more articles on functional data analysis have been published in genome-wide association analysis [26,31,32,41,42,43,44]. The association analysis method based on the functional linear regression model can not only estimate the additive and dominant effects of genes, but also estimate the epistasis effects of genes [31,32], and extended to the study of dynamic development and multiple traits, Li [45] proposed a longitudinal functional data association test (LFDAT) based on the function–function regression model, which can provide a feasible method for studying the formation and expression of longitudinal traits. Li [46] put forward an integrative functional linear model for GWAS with multiple traits, which effectively accommodates the high dimensionality of SNPs and correlation among multiple traits. However, current analysis methods can develop only based on SNP gene region, it is impossible to further study whether SNP inside gene regions are associated with phenotypic traits.

In practice, gene loci are linkage disequilibrium with each other. The simulation results of this paper also show that most of the indictors under the linkage disequilibrium are better than that based on the linkage equilibrium, especially since the power of linkage disequilibrium is much higher than that of linkage equilibrium. Therefore, there is a considerable gap between the simulation results with a measure of 0.2 linkage disequilibrium and linkage equilibrium, so this paper does not further explore the simulation of linkage disequilibrium with a higher measure.

**Figure 5:**
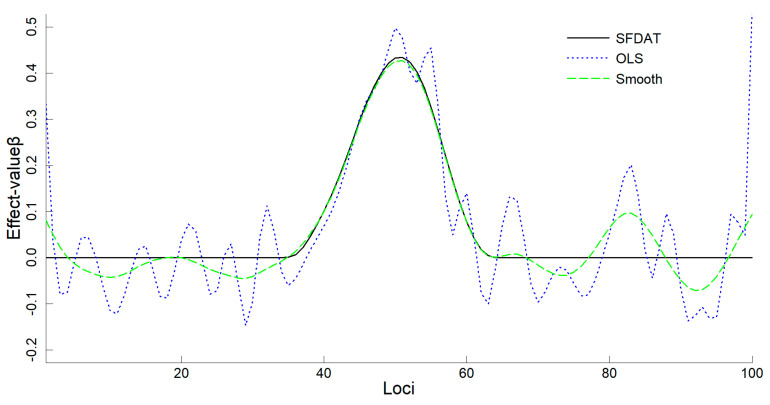
Effect functions $\hat{\beta }\left(t\right)$ estimated by SFDAT, OLS and Smooth methods. Solid line represents the SFDAT, dot line represents the OLS and dashed line represents the Smooth.

Figure 5 shows the effect of function $\hat{\beta }(t)$ curve by OLS, Smooth and SFDAT methods. Firstly, from Figure 5, it can be seen that the effect function of the OLS and Smooth methods $\hat{\beta }(t)$ have frequent fluctuations. Compared to the OLS and Smooth methods, the estimated effect function $\hat{\beta }(t)$ by the SFDAT method could smooth the effect value which real effect values are zero to around zero and still retain the non-zero part of the real effect. Combining the CSR and DL of SFDAT, it shows that the smoothing function can indeed remove some “noise”, which also explains the reason why false associations are detected by the OLS and Smooth methods. Secondly, there are similar results for non-zero genetic effects estimated to use the Smooth and SFDAT methods. This shows that the compression capability of the SFDAT method can be regarded as the further compression of the effect value close to zero on the basis of the smooth estimation results, while retaining the non-zero effect. Therefore, if the compression parameter is small and there are no effect values worth compressing in the gene region, the estimated effect function of the SFDAT method should be consistent with that of the Smooth method. It is the reason why the Smooth and SFDAT methods have similar power, ISE_0_, ISE_1_, PMSE and the type I error rate in computer simulations, but relatively speaking, SFDAT still has a higher accuracy. In addition, in the application of real data, QTL analysis often takes a lot of time. Therefore, during GWAS, all gene regions can be quickly scanned through SFDAT to find the gene regions where significant SNP loci are located, and then QTL analysis can be performed on these gene regions to accurately locate the positions of significant SNPs, which can save a lot of time and improve efficiency beyond all doubt.

It must be pointed out that Luo et al. [26] proposed a functional linear regression model for QTL association analysis based on next-generation high-throughput sequencing (NGS), which used the functional linear regression model method (FLM). The smoothed functional linear model (SFLM) and eight other statistical methods (WSS, VT, RVT1, RVT2, PCA regression, multiple regression, simple regression and SKAT) were compared in six cases (uniformity of effects means the same direction; heterogeneity of effects means different directions; only low-frequency variants; all variants; different proportions of causal variants; different proportions of variants included). It found that the FLM method and SFLM method are similar in each case, but they are obviously superior to other methods, including collapsing-based methods RVT1, RVT2, kernel-based methods SKAT. The FLM and SFLM proposed by Luo et al. [26] differ from our OLS and Smooth methods in loss function Loss(β,μ). The first term of loss function Loss(β,μ) for the OLS, Smooth and SFDAT methods is $\sum_{i=1}^{n}[y_{i}-\mu -\int_{0}^{T}X_{i}(t)\beta (t)dt{]}^{2}$ divided by the sample size n, the first term of loss function Loss(β,μ) for the FLM and SFLM methods is not divided by the sample size n. However, the idea of FLM and SFLM is similar to that of the OLS, Smooth and SFDAT methods. SFLM and Smooth method are only an adjustment among parameters. Although the OLS, Smooth and SFDAT methods have not been compared with traditional methods, the SFDAT method should be a good method according to Luo et al. [26] and our simulation's results. It should be noted that the SFDAT method is only based on a single-gene region for a one-by-one search in this paper. In fact, we can extend the method to multiple-gene regions detection which is our next research direction.

In the application of the functional linear regression model for gene association analysis, we mainly convert the functional linear regression model into the classical linear regression model for parameter estimation and statistical tests. However, we know that gene variants have common variants and rare variants so there are unique methods for rare variant detection [47]. This is especially true for statistical test problems of functional linear regression model which has also been proposed by some scholars [48]. Therefore, how to better perform statistical tests on gene association analysis using the functional linear regression models remains to be further studied.

## 6. Conclusions

In this paper, to address the problem of the sparsity of gene regions in association analysis, we develop a functional linear model with a SCAD penalty to genome-wide association analysis and propose a sparse functional data association analysis test (SFDAT) method. SFDAT can compress unassociated SNP loci in gene regions without reducing the power too much, and reduces false positives in association analysis. Our simulation studies and real data analysis also show that SFDAT can detect non-effect SNP loci more accurately and compress their coefficients to zero compared to OLS and Smooth, while maintaining higher power and lower false positives. Thus, SFDAT is a powerful tool for GWAS using next-generation sequencing data.

## Figures and Tables

**Table 1:** The power of association analysis of simulated quantitative trait for linkage equilibrium and linkage disequilibrium based on SFDAT, OLS and Smooth at significance level of 0.01.

Associated Variants Proportion	*c*	Negative Effect Proportion	*r* ^2^	Common Variants			Low-Frequency Variants			Rare Variants			Mixed Variants		
				SFDAT	OLS	Smooth	SFDAT	OLS	Smooth	SFDAT	OLS	Smooth	SFDAT	OLS	Smooth
1/180	3	0	0	0.274	0.293	0.285	0.203	0.237	0.203	0.039	0.108	0.039	0.034	0.089	0.036
			0.2	1.000	1.000	1.000	0.999	0.999	0.999	0.318	0.438	0.319	0.993	0.995	0.994
		0.2	0	0.217	0.233	0.228	0.163	0.195	0.163	0.036	0.080	0.037	0.046	0.107	0.046
			0.2	0.990	0.995	0.995	0.833	0.857	0.836	0.141	0.238	0.142	0.821	0.837	0.831
		0.4	0	0.193	0.210	0.199	0.145	0.177	0.146	0.026	0.077	0.026	0.046	0.092	0.046
			0.2	0.239	0.267	0.251	0.278	0.319	0.280	0.060	0.119	0.061	0.281	0.298	0.288
	5	0	0	0.528	0.542	0.534	0.452	0.491	0.453	0.147	0.229	0.147	0.159	0.269	0.159
			0.2	1.000	1.000	1.000	1.000	1.000	1.000	0.721	0.806	0.723	0.998	0.998	0.998
		0.2	0	0.466	0.483	0.472	0.432	0.480	0.432	0.132	0.232	0.133	0.125	0.219	0.127
			0.2	1.000	1.000	1.000	0.985	0.986	0.985	0.415	0.542	0.419	0.953	0.955	0.954
		0.4	0	0.443	0.456	0.446	0.403	0.459	0.403	0.096	0.199	0.097	0.123	0.214	0.123
			0.2	0.589	0.617	0.604	0.617	0.650	0.617	0.187	0.308	0.189	0.562	0.573	0.564
	7	0	0	0.636	0.648	0.638	0.614	0.654	0.617	0.250	0.349	0.250	0.240	0.358	0.241
			0.2	1.000	1.000	1.000	1.000	1.000	1.000	0.857	0.902	0.858	1.000	1.000	1.000
		0.2	0	0.604	0.626	0.612	0.577	0.620	0.577	0.225	0.352	0.225	0.209	0.311	0.210
			0.2	1.000	1.000	1.000	0.999	1.000	0.999	0.588	0.701	0.590	0.970	0.971	0.970
		0.4	0	0.568	0.581	0.569	0.517	0.566	0.517	0.180	0.298	0.180	0.197	0.322	0.197
			0.2	0.757	0.763	0.760	0.753	0.799	0.753	0.354	0.499	0.357	0.694	0.706	0.697
1/90	3	0	0	0.618	0.638	0.630	0.590	0.624	0.591	0.178	0.286	0.179	0.162	0.267	0.163
			0.2	1.000	1.000	1.000	1.000	1.000	1.000	0.922	0.953	0.924	1.000	1.000	1.000
		0.2	0	0.485	0.495	0.490	0.428	0.479	0.430	0.103	0.223	0.103	0.119	0.232	0.121
			0.2	1.000	1.000	1.000	1.000	1.000	1.000	0.565	0.680	0.568	0.998	0.998	0.998
		0.4	0	0.463	0.479	0.465	0.384	0.450	0.384	0.102	0.192	0.104	0.104	0.187	0.104
			0.2	0.807	0.821	0.815	0.713	0.751	0.715	0.196	0.336	0.196	0.650	0.669	0.656
	5	0	0	0.852	0.859	0.854	0.850	0.869	0.850	0.472	0.595	0.473	0.472	0.616	0.472
			0.2	1.000	1.000	1.000	1.000	1.000	1.000	0.996	0.997	0.996	1.000	1.000	1.000
		0.2	0	0.781	0.793	0.784	0.742	0.775	0.742	0.343	0.464	0.344	0.338	0.496	0.338
			0.2	1.000	1.000	1.000	1.000	1.000	1.000	0.869	0.910	0.869	0.998	0.998	0.998
		0.4	0	0.698	0.707	0.700	0.672	0.713	0.672	0.316	0.449	0.318	0.287	0.436	0.287
			0.2	0.964	0.969	0.966	0.922	0.936	0.922	0.532	0.683	0.534	0.846	0.853	0.847
	7	0	0	0.920	0.922	0.920	0.920	0.931	0.920	0.628	0.748	0.629	0.643	0.757	0.645
			0.2	1.000	1.000	1.000	1.000	1.000	1.000	0.999	1.000	1.000	1.000	1.000	1.000
		0.2	0	0.851	0.855	0.851	0.851	0.870	0.851	0.514	0.642	0.514	0.508	0.642	0.508
			0.2	1.000	1.000	1.000	1.000	1.000	1.000	0.958	0.971	0.958	1.000	1.000	1.000
		0.4	0	0.809	0.813	0.809	0.747	0.777	0.747	0.456	0.590	0.458	0.463	0.603	0.463
			0.2	0.983	0.987	0.984	0.966	0.975	0.966	0.750	0.845	0.752	0.921	0.925	0.921
1/45	3	0	0	0.949	0.950	0.949	0.945	0.955	0.945	0.607	0.742	0.607	0.596	0.725	0.599
			0.2	1.000	1.000	1.000	1.000	1.000	1.000	1.000	1.000	1.000	1.000	1.000	1.000
		0.2	0	0.812	0.815	0.813	0.802	0.837	0.802	0.399	0.549	0.400	0.396	0.564	0.398
			0.2	1.000	1.000	1.000	1.000	1.000	1.000	0.985	0.992	0.985	1.000	1.000	1.000
		0.4	0	0.712	0.724	0.712	0.690	0.729	0.690	0.286	0.441	0.286	0.274	0.430	0.276
			0.2	0.998	0.998	0.998	0.973	0.980	0.973	0.575	0.711	0.575	0.928	0.930	0.929
	5	0	0	0.997	0.997	0.997	0.995	0.995	0.995	0.919	0.949	0.919	0.910	0.947	0.910
			0.2	1.000	1.000	1.000	1.000	1.000	1.000	1.000	1.000	1.000	1.000	1.000	1.000
		0.2	0	0.935	0.938	0.935	0.942	0.952	0.942	0.740	0.843	0.742	0.720	0.816	0.720
			0.2	1.000	1.000	1.000	1.000	1.000	1.000	0.999	1.000	0.999	1.000	1.000	1.000
		0.4	0	0.859	0.868	0.859	0.862	0.886	0.862	0.587	0.722	0.587	0.605	0.748	0.606
			0.2	1.000	1.000	1.000	0.992	0.994	0.992	0.869	0.928	0.870	0.970	0.975	0.970
	7	0	0	0.997	0.997	0.997	0.999	0.999	0.999	0.963	0.979	0.963	0.959	0.978	0.959
			0.2	1.000	1.000	1.000	1.000	1.000	1.000	1.000	1.000	1.000	1.000	1.000	1.000
		0.2	0	0.970	0.971	0.970	0.969	0.974	0.969	0.855	0.914	0.855	0.841	0.906	0.841
			0.2	1.000	1.000	1.000	1.000	1.000	1.000	0.999	0.999	0.999	1.000	1.000	1.000
		0.4	0	0.892	0.895	0.892	0.901	0.923	0.901	0.716	0.822	0.716	0.727	0.853	0.727
			0.2	1.000	1.000	1.000	0.997	0.997	0.997	0.946	0.975	0.946	0.982	0.984	0.982

Note: *r^2^* represents the measure of linkage disequilibrium, *r^2^* equals to 0 means there is linkage equilibrium between SNP.

**Table 2:** The means and standard errors (in the parenthesis) of ISE_0_ for association analysis of simulated quantitative trait based on SFDAT, OLS and Smooth on linkage equilibrium and linkage disequilibrium.

Associated Variants Proportion	*c*	Negative Effect Proportion	*r* ^2^	Common Variants			Low-Frequency Variants			Rare Variants			Mix Variants		
				SFDAT	OLS	Smooth	SFDAT	OLS	Smooth	SFDAT	OLS	Smooth	SFDAT	OLS	Smooth
1/180	3	0	0	0.068	0.071	0.069	0.379	0.414	0.380	1.431	1.810	1.445	1.418	1.789	1.433
				(0.027)	(0.027)	(0.026)	(0.121)	(0.134)	(0.120)	(0.468)	(0.549)	(0.456)	(0.451)	(0.540)	(0.437)
			0.2	0.061	0.064	0.062	0.402	0.440	0.404	1.553	1.971	1.564	0.097	0.102	0.098
				(0.019)	(0.018)	(0.017)	(0.125)	(0.136)	(0.125)	(0.503)	(0.605)	(0.495)	(0.032)	(0.032)	(0.031)
		0.2	0	0.066	0.068	0.067	0.379	0.413	0.381	1.414	1.764	1.425	1.462	1.834	1.473
				(0.025)	(0.025)	(0.024)	(0.124)	(0.134)	(0.122)	(0.467)	(0.553)	(0.458)	(0.492)	(0.606)	(0.485)
			0.2	0.061	0.064	0.063	0.394	0.431	0.395	1.566	1.982	1.577	0.095	0.100	0.097
				(0.020)	(0.019)	(0.019)	(0.127)	(0.138)	(0.125)	(0.514)	(0.613)	(0.505)	(0.030)	(0.031)	(0.029)
		0.4	0	0.065	0.068	0.067	0.377	0.411	0.379	1.395	1.763	1.412	1.435	1.800	1.445
				(0.027)	(0.025)	(0.026)	(0.122)	(0.131)	(0.121)	(0.476)	(0.567)	(0.464)	(0.489)	(0.588)	(0.481)
			0.2	0.059	0.063	0.062	0.399	0.435	0.401	1.573	2.004	1.588	0.096	0.101	0.098
				(0.020)	(0.019)	(0.018)	(0.123)	(0.133)	(0.122)	(0.524)	(0.626)	(0.509)	(0.032)	(0.032)	(0.030)
	5	0	0	0.102	0.105	0.103	0.549	0.595	0.549	1.809	2.252	1.816	1.818	2.275	1.827
				(0.048)	(0.049)	(0.048)	(0.191)	(0.215)	(0.190)	(0.642)	(0.773)	(0.637)	(0.672)	(0.862)	(0.673)
			0.2	0.084	0.087	0.085	0.581	0.632	0.582	2.039	2.571	2.045	0.132	0.138	0.133
				(0.025)	(0.025)	(0.025)	(0.183)	(0.202)	(0.183)	(0.659)	(0.791)	(0.656)	(0.044)	(0.046)	(0.044)
		0.2	0	0.103	0.106	0.104	0.569	0.621	0.570	1.828	2.295	1.837	1.834	2.293	1.841
				(0.051)	(0.052)	(0.051)	(0.204)	(0.227)	(0.203)	(0.669)	(0.822)	(0.662)	(0.663)	(0.825)	(0.660)
			0.2	0.085	0.088	0.086	0.584	0.634	0.585	2.032	2.566	2.041	0.137	0.142	0.137
				(0.027)	(0.027)	(0.026)	(0.184)	(0.203)	(0.183)	(0.673)	(0.820)	(0.666)	(0.046)	(0.049)	(0.046)
		0.4	0	0.101	0.104	0.102	0.564	0.614	0.565	1.815	2.264	1.823	1.817	2.275	1.827
				(0.046)	(0.047)	(0.046)	(0.198)	(0.222)	(0.198)	(0.635)	(0.774)	(0.627)	(0.652)	(0.801)	(0.646)
			0.2	0.085	0.088	0.086	0.585	0.637	0.585	1.994	2.516	2.003	0.132	0.137	0.133
				(0.028)	(0.028)	(0.027)	(0.182)	(0.202)	(0.181)	(0.651)	(0.794)	(0.644)	(0.044)	(0.046)	(0.044)
	7	0	0	0.131	0.134	0.131	0.714	0.222	0.714	2.169	2.707	2.174	2.165	2.721	2.172
				(0.066)	(0.069)	(0.066)	(0.265)	(0.309)	(0.265)	(0.822)	(1.070)	(0.818)	(0.773)	(1.000)	(0.770)
			0.2	0.106	0.109	0.107	0.730	0.795	0.730	2.404	3.027	2.409	0.167	0.173	0.167
				(0.033)	(0.033)	(0.032)	(0.218)	(0.243)	(0.218)	(0.815)	(1.003)	(0.810)	(0.058)	(0.061)	(0.058)
		0.2	0	0.132	0.135	0.132	0.737	0.800	0.738	2.173	2.726	2.179	2.158	2.698	2.165
				(0.065)	(0.068)	(0.065)	(0.280)	(0.319)	(0.280)	(0.822)	(1.076)	(0.817)	(0.805)	(1.024)	(0.800)
			0.2	0.106	0.109	0.107	0.746	0.813	0.746	2.415	3.055	2.421	0.167	0.173	0.168
				(0.034)	(0.035)	(0.033)	(0.242)	(0.267)	(0.242)	(0.826)	(1.025)	(0.821)	(0.058)	(0.062)	(0.058)
		0.4	0	0.129	0.132	0.129	0.710	0.772	0.710	2.109	2.636	2.117	2.167	2.717	2.174
				(0.064)	(0.066)	(0.063)	(0.254)	(0.283)	(0.253)	(0.779)	(0.968)	(0.775)	(0.810)	(1.064)	(0.805)
			0.2	0.106	0.109	0.107	0.753	0.821	0.753	2.448	3.075	2.455	0.168	0.174	0.168
				(0.033)	(0.034)	(0.033)	(0.240)	(0.272)	(0.239)	(0.867)	(1.048)	(0.863)	(0.057)	(0.060)	(0.057)
1/90	3	0	0	0.099	0.101	0.099	0.548	0.596	0.548	1.791	2.235	1.796	1.782	2.221	1.789
				(0.039)	(0.040)	(0.038)	(0.171)	(0.191)	(0.171)	(0.617)	(0.755)	(0.613)	(0.566)	(0.685)	(0.562)
			0.2	0.081	0.084	0.082	0.554	0.603	0.554	1.989	2.520	1.992	0.130	0.135	0.130
				(0.024)	(0.025)	(0.024)	(0.171)	(0.184)	(0.171)	(0.642)	(0.786)	(0.640)	(0.039)	(0.041)	(0.039)
		0.2	0	0.097	0.099	0.098	0.534	0.579	0.534	1.787	2.230	1.793	1.780	2.222	1.788
				(0.037)	(0.038)	(0.036)	(0.166)	(0.185)	(0.166)	(0.557)	(0.674)	(0.554)	(0.583)	(0.712)	(0.578)
			0.2	0.082	0.085	0.083	0.550	0.600	0.551	1.965	2.491	1.973	0.128	0.134	0.129
				(0.025)	(0.025)	(0.024)	(0.165)	(0.181)	(0.165)	(0.641)	(0.779)	(0.632)	(0.042)	(0.044)	(0.041)
		0.4	0	0.099	0.101	0.099	0.555	0.603	0.555	1.803	2.242	1.809	1.784	2.226	1.792
				(0.035)	(0.036)	(0.035)	(0.171)	(0.187)	(0.171)	(0.589)	(0.716)	(0.584)	(0.592)	(0.709)	(0.588)
			0.2	0.082	0.085	0.083	0.576	0.626	0.576	1.969	2.482	1.976	0.130	0.135	0.130
				(0.025)	(0.025)	(0.024)	(0.174)	(0.188)	(0.174)	(0.617)	(0.734)	(0.612)	(0.042)	(0.043)	(0.041)
	5	0	0	0.165	0.169	0.166	0.901	0.975	0.901	2.601	3.228	2.605	2.601	3.239	2.604
				(0.068)	(0.070)	(0.067)	(0.289)	(0.320)	(0.289)	(0.906)	(1.135)	(0.904)	(0.929)	(1.131)	(0.926)
			0.2	0.130	0.133	0.130	0.922	1.004	0.922	2.867	3.620	2.870	0.204	0.211	0.204
				(0.039)	(0.040)	(0.039)	(0.274)	(0.300)	(0.274)	(0.907)	(1.111)	(0.905)	(0.068)	(0.073)	(0.068)
		0.2	0	0.170	0.173	0.170	0.896	0.973	0.896	2.567	3.199	2.570	2.595	3.234	2.599
				(0.071)	(0.074)	(0.071)	(0.301)	(0.343)	(0.301)	(0.968)	(1.227)	(0.966)	(0.882)	(1.135)	(0.881)
			0.2	0.129	0.132	0.129	0.922	1.004	0.922	2.862	3.605	2.865	0.202	0.209	0.202
				(0.040)	(0.040)	(0.039)	(0.284)	(0.312)	(0.284)	(0.934)	(1.140)	(0.933)	(0.066)	(0.070)	(0.066)
		0.4	0	0.166	0.169	0.166	0.921	0.999	0.921	2.638	3.280	2.642	2.598	3.244	2.603
				(0.069)	(0.071)	(0.069)	(0.297)	(0.336)	(0.297)	(0.896)	(1.125)	(0.892)	(0.934)	(1.139)	(0.929)
			0.2	0.130	0.133	0.131	0.934	1.016	0.934	2.908	3.666	2.912	0.200	0.207	0.200
				(0.040)	(0.041)	(0.040)	(0.284)	(0.309)	(0.284)	(0.950)	(1.184)	(0.949)	(0.064)	(0.067)	(0.064)
	7	0	0	0.223	0.227	0.223	1.217	1.322	1.217	3.287	4.095	3.290	3.298	4.083	3.300
				(0.097)	(0.101)	(0.097)	(0.404)	(0.455)	(0.404)	(1.220)	(1.563)	(1.220)	(1.207)	(1.465)	(1.206)
			0.2	0.168	0.172	0.168	1.235	1.344	1.235	3.606	4.528	3.607	0.265	0.274	0.265
				(0.053)	(0.054)	(0.053)	(0.372)	(0.409)	(0.372)	(1.250)	(1.540)	(1.250)	(0.088)	(0.093)	(0.088)
		0.2	0	0.225	0.229	0.225	1.211	1.309	1.211	3.303	4.096	3.305	3.303	4.111	3.306
				(0.097)	(0.100)	(0.097)	(0.387)	(0.429)	(0.387)	(1.157)	(1.429)	(1.156)	(1.238)	(1.560)	(1.237)
			0.2	0.170	0.173	0.170	1.246	1.353	1.246	3.705	4.664	3.706	0.267	0.276	0.267
				(0.053)	(0.055)	(0.053)	(0.385)	(0.422)	(0.385)	(1.270)	(1.567)	(1.269)	(0.086)	(0.091)	(0.086)
		0.4	0	0.224	0.228	0.224	1.215	1.316	1.215	3.312	4.110	3.314	3.348	4.154	3.349
				(0.092)	(0.094)	(0.092)	(0.424)	(0.471)	(0.424)	(1.174)	(1.492)	(1.173)	(1.196)	(1.529)	(1.196)
			0.2	0.172	0.176	0.172	1.276	1.387	1.276	3.773	4.740	3.776	0.267	0.276	0.267
				(0.050)	(0.051)	(0.050)	(0.401)	(0.445)	(0.400)	(1.190)	(1.486)	(1.188)	(0.087)	(0.092)	(0.087)
1/45	3	0	0	0.154	0.157	0.154	0.841	0.914	0.841	2.476	3.083	2.478	2.487	3.098	2.489
				(0.054)	(0.055)	(0.054)	(0.259)	(0.283)	(0.259)	(0.807)	(0.978)	(0.807)	(0.794)	(0.972)	(0.795)
			0.2	0.122	0.125	0.122	0.863	0.941	0.863	2.737	3.443	2.738	0.198	0.204	0.198
				(0.037)	(0.038)	(0.037)	(0.251)	(0.275)	(0.251)	(0.887)	(1.069)	(0.887)	(0.062)	(0.066)	(0.062)
		0.2	0	0.157	0.160	0.157	0.854	0.926	0.854	2.522	3.134	2.526	2.489	3.108	2.491
				(0.056)	(0.058)	(0.056)	(0.258)	(0.277)	(0.258)	(0.831)	(0.977)	(0.828)	(0.818)	(0.995)	(0.817)
			0.2	0.123	0.126	0.123	0.879	0.956	0.879	2.789	3.529	2.790	0.195	0.202	0.195
				(0.034)	(0.035)	(0.034)	(0.254)	(0.281)	(0.254)	(0.894)	(1.103)	(0.893)	(0.063)	(0.066)	(0.063)
		0.4	0	0.160	0.164	0.161	0.870	0.944	0.870	2.523	3.146	2.527	2.475	3.095	2.480
				(0.059)	(0.060)	(0.058)	(0.262)	(0.290)	(0.262)	(0.790)	(0.969)	(0.787)	(0.779)	(0.953)	(0.776)
			0.2	0.123	0.125	0.123	0.896	0.973	0.896	2.815	3.540	2.818	0.195	0.203	0.196
				(0.037)	(0.037)	(0.037)	(0.264)	(0.289)	(0.264)	(0.887)	(1.075)	(0.886)	(0.059)	(0.061)	(0.058)
	5	0	0	0.292	0.297	0.292	1.581	1.717	1.581	4.125	5.089	4.125	4.133	5.146	4.133
				(0.107)	(0.110)	(0.107)	(0.509)	(0.562)	(0.509)	(1.443)	(1.717)	(1.443)	(1.372)	(1.728)	(1.372)
			0.2	0.219	0.224	0.219	1.609	1.752	1.609	4.661	5.852	4.661	0.353	0.365	0.353
				(0.067)	(0.068)	(0.067)	(0.470)	(0.516)	(0.470)	(1.419)	(1.737)	(1.419)	(0.114)	(0.122)	(0.114)
		0.2	0	0.298	0.304	0.298	1.617	1.758	1.617	4.144	5.163	4.146	4.057	5.043	4.058
				(0.108)	(0.112)	(0.108)	(0.512)	(0.565)	(0.512)	(1.398)	(1.732)	(1.397)	(1.341)	(1.667)	(1.341)
			0.2	0.217	0.222	0.217	1.631	1.772	1.631	4.687	5.839	4.687	0.346	0.358	0.346
				(0.065)	(0.067)	(0.065)	(0.489)	(0.524)	(0.489)	(1.549)	(1.844)	(1.549)	(0.109)	(0.115)	(0.109)
		0.4	0	0.296	0.302	0.296	1.598	1.733	1.598	4.121	5.095	4.122	4.193	5.172	4.194
				(0.111)	(0.114)	(0.111)	(0.504)	(0.562)	(0.504)	(1.368)	(1.717)	(1.367)	(1.408)	(1.699)	(1.408)
			0.2	0.218	0.222	0.218	1.655	1.799	1.655	4.659	5.841	4.659	0.342	0.354	0.342
				(0.063)	(0.064)	(0.063)	(0.487)	(0.530)	(0.487)	(1.505)	(1.844)	(1.504)	(0.109)	(0.118)	(0.109)
	7	0	0	0.404	0.412	0.404	2.161	2.344	2.161	5.402	6.695	5.401	5.367	6.695	5.368
				(0.145)	(0.148)	(0.145)	(0.637)	(0.700)	(0.637)	(1.857)	(2.245)	(1.858)	(1.771)	(2.208)	(1.771)
			0.2	0.296	0.302	0.296	2.216	2.412	2.216	6.211	7.795	6.211	0.480	0.497	0.480
				(0.086)	(0.088)	(0.086)	(0.652)	(0.710)	(0.652)	(1.915)	(2.299)	(1.915)	(0.158)	(0.168)	(0.158)
		0.2	0	0.409	0.417	0.409	2.209	2.402	2.209	5.446	6.791	5.447	5.516	6.813	5.516
				(0.155)	(0.160)	(0.155)	(0.713)	(0.784)	(0.713)	(1.892)	(2.382)	(1.893)	(1.864)	(2.322)	(1.864)
			0.2	0.298	0.304	0.298	2.281	2.474	2.281	6.234	7.830	6.234	0.472	0.488	0.472
				(0.087)	(0.089)	(0.087)	(0.659)	(0.718)	(0.659)	(1.936)	(2.375)	(1.936)	(0.142)	(0.151)	(0.142)
		0.4	0	0.408	0.415	0.408	2.213	2.394	2.213	5.551	6.877	5.552	5.510	6.868	5.511
				(0.158)	(0.163)	(0.158)	(0.736)	(0.804)	(0.736)	(1.922)	(2.443)	(1.922)	(1.832)	(2.307)	(1.831)
			0.2	0.296	0.302	0.296	2.318	2.526	2.318	6.384	8.051	6.385	0.471	0.487	0.471
				(0.088)	(0.090)	(0.088)	(0.678)	(0.744)	(0.678)	(2.048)	(2.528)	(2.049)	(0.152)	(0.160)	(0.152)

Note: Each data in the table are multiplied by 10^−3^. Each data unit has an average value of ISE_0_ on top and a standard deviation of ISE_0_ in parentheses below, *r^2^* represents the measure of linkage disequilibrium, *r^2^* equals to 0 means there is linkage equilibrium between SNP.

**Table 3:** The means and standard errors (in the parenthesis) of ISE_1_ for association analysis of simulated quantitative trait based on SFDAT, OLS and Smooth on linkage equilibrium and linkage disequilibrium.

Associated Variants Proportion	*c*	Negative Effect Proportion	*r* ^2^	Common Variants			Low-Frequency Variants			Rare Variants			Mix Variants		
				SFDAT	OLS	Smooth	SFDAT	OLS	Smooth	SFDAT	OLS	Smooth	SFDAT	OLS	Smooth
1/180	3	0	0	0.018	0.018	0.018	0.011	0.011	0.011	0.029	0.029	0.029	0.027	0.027	0.027
				(0.012)	(0.012)	(0.012)	(0.007)	(0.007)	(0.007)	(0.022)	(0.023)	(0.022)	(0.020)	(0.021)	(0.020)
			0.2	0.019	0.019	0.019	0.013	0.013	0.013	0.056	0.057	0.056	0.150	0.150	0.150
				(0.013)	(0.013)	(0.013)	(0.008)	(0.008)	(0.008)	(0.052)	(0.054)	(0.052)	(0.098)	(0.098)	(0.098)
		0.2	0	0.102	0.102	0.102	0.560	0.559	0.560	1.293	1.284	1.292	1.302	1.292	1.301
				(0.043)	(0.043)	(0.043)	(0.076)	(0.076)	(0.076)	(0.188)	(0.189)	(0.188)	(0.190)	(0.190)	(0.190)
			0.2	0.112	0.112	0.112	0.569	0.567	0.569	1.405	1.397	1.404	0.448	0.448	0.448
				(0.050)	(0.050)	(0.050)	(0.085)	(0.086)	(0.085)	(0.304)	(0.307)	(0.305)	(0.268)	(0.268)	(0.268)
		0.4	0	0.147	0.147	0.147	0.839	0.837	0.839	1.942	1.928	1.941	1.939	1.927	1.939
				(0.057)	(0.057)	(0.057)	(0.098)	(0.098)	(0.098)	(0.250)	(0.252)	(0.250)	(0.237)	(0.237)	(0.237)
			0.2	0.154	0.154	0.154	0.846	0.845	0.846	2.101	2.089	2.101	0.596	0.596	0.596
				(0.060)	(0.060)	(0.060)	(0.105)	(0.106)	(0.105)	(0.378)	(0.382)	(0.378)	(0.321)	(0.321)	(0.321)
	5	0	0	0.040	0.040	0.040	0.023	0.023	0.023	0.061	0.062	0.061	0.057	0.058	0.057
				(0.028)	(0.028)	(0.028)	(0.014)	(0.014)	(0.014)	(0.047)	(0.048)	(0.047)	(0.044)	(0.044)	(0.043)
			0.2	0.043	0.043	0.043	0.026	0.027	0.026	0.115	0.117	0.115	0.329	0.329	0.329
				(0.027)	(0.027)	(0.027)	(0.016)	(0.016)	(0.016)	(0.109)	(0.111)	(0.109)	(0.212)	(0.212)	(0.212)
		0.2	0	0.228	0.228	0.228	1.219	1.216	1.219	2.820	2.802	2.819	2.790	2.771	2.790
				(0.099)	(0.099)	(0.099)	(0.166)	(0.166)	(0.166)	(0.422)	(0.419)	(0.422)	(0.418)	(0.419)	(0.418)
			0.2	0.234	0.233	0.233	1.230	1.227	1.230	3.043	3.027	3.042	0.955	0.955	0.955
				(0.105)	(0.105)	(0.105)	(0.184)	(0.184)	(0.184)	(0.650)	(0.656)	(0.650)	(0.558)	(0.558)	(0.558)
		0.4	0	0.314	0.314	0.314	1.798	1.794	1.798	4.155	4.127	4.154	4.162	4.134	4.161
				(0.123)	(0.123)	(0.123)	(0.210)	(0.211)	(0.210)	(0.536)	(0.539)	(0.536)	(0.532)	(0.535)	(0.532)
			0.2	0.335	0.335	0.335	1.823	1.819	1.822	4.501	4.477	4.501	1.260	1.260	1.260
				(0.128)	(0.128)	(0.128)	(0.225)	(0.226)	(0.225)	(0.803)	(0.808)	(0.804)	(0.692)	(0.692)	(0.692)
	7	0	0	0.055	0.055	0.055	0.032	0.032	0.032	0.084	0.085	0.084	0.082	0.084	0.082
				(0.037)	(0.037)	(0.037)	(0.019)	(0.019)	(0.019)	(0.063)	(0.065)	(0.063)	(0.063)	(0.064)	(0.063)
			0.2	0.060	0.060	0.060	0.040	0.040	0.040	0.174	0.178	0.174	0.475	0.475	0.475
				(0.041)	(0.041)	(0.041)	(0.022)	(0.023)	(0.022)	(0.168)	(0.171)	(0.168)	(0.302)	(0.302)	(0.302)
		0.2	0	0.328	0.328	0.328	1.767	1.763	1.767	4.090	4.063	4.090	4.080	4.053	4.079
				(0.143)	(0.143)	(0.143)	(0.239)	(0.239)	(0.239)	(0.606)	(0.607)	(0.605)	(0.605)	(0.602)	(0.604)
			0.2	0.345	0.344	0.345	1.786	1.782	1.786	4.425	4.402	4.425	1.377	1.376	1.377
				(0.150)	(0.150)	(0.150)	(0.270)	(0.272)	(0.270)	(0.967)	(0.976)	(0.967)	(0.813)	(0.813)	(0.813)
		0.4	0	0.465	0.464	0.465	2.637	2.630	2.637	6.100	6.059	6.099	6.080	6.039	6.080
				(0.180)	(0.180)	(0.180)	(0.308)	(0.308)	(0.308)	(0.779)	(0.779)	(0.779)	(0.755)	(0.759)	(0.754)
			0.2	0.479	0.478	0.478	2.652	2.646	2.652	6.543	6.508	6.542	1.842	1.841	1.842
				(0.181)	(0.181)	(0.181)	(0.321)	(0.323)	(0.321)	(1.163)	(1.171)	(1.162)	(1.007)	(1.007)	(1.007)
1/90	3	0	0	0.018	0.018	0.018	0.011	0.011	0.011	0.029	0.030	0.029	0.028	0.029	0.028
				(0.008)	(0.008)	(0.008)	(0.004)	(0.005)	(0.004)	(0.014)	(0.015)	(0.014)	(0.015)	(0.016)	(0.015)
			0.2	0.020	0.020	0.020	0.013	0.013	0.013	0.056	0.058	0.056	0.151	0.151	0.151
				(0.009)	(0.009)	(0.009)	(0.005)	(0.005)	(0.005)	(0.036)	(0.037)	(0.036)	(0.064)	(0.064)	(0.064)
		0.2	0	0.094	0.094	0.094	0.502	0.501	0.502	1.160	1.153	1.160	1.165	1.158	1.164
				(0.029)	(0.029)	(0.029)	(0.050)	(0.050)	(0.050)	(0.128)	(0.129)	(0.128)	(0.127)	(0.128)	(0.127)
			0.2	0.099	0.099	0.099	0.509	0.508	0.509	1.270	1.264	1.270	0.415	0.414	0.414
				(0.032)	(0.032)	(0.032)	(0.055)	(0.055)	(0.055)	(0.197)	(0.199)	(0.197)	(0.171)	(0.171)	(0.171)
		0.4	0	0.134	0.134	0.134	0.748	0.746	0.748	1.730	1.719	1.730	1.723	1.712	1.722
				(0.037)	(0.037)	(0.037)	(0.063)	(0.063)	(0.063)	(0.160)	(0.161)	(0.160)	(0.150)	(0.151)	(0.150)
			0.2	0.139	0.139	0.139	0.759	0.757	0.759	1.859	1.849	1.859	0.543	0.543	0.543
				(0.039)	(0.039)	(0.039)	(0.065)	(0.065)	(0.065)	(0.245)	(0.248)	(0.245)	(0.205)	(0.205)	(0.205)
	5	0	0	0.038	0.038	0.038	0.022	0.022	0.022	0.058	0.059	0.058	0.059	0.061	0.059
				(0.017)	(0.017)	(0.017)	(0.009)	(0.009)	(0.009)	(0.029)	(0.030)	(0.029)	(0.030)	(0.031)	(0.030)
			0.2	0.043	0.043	0.043	0.027	0.027	0.027	0.125	0.128	0.125	0.319	0.319	0.319
				(0.018)	(0.018)	(0.018)	(0.011)	(0.011)	(0.011)	(0.079)	(0.080)	(0.079)	(0.140)	(0.140)	(0.140)
		0.2	0	0.204	0.204	0.204	1.080	1.078	1.080	2.499	2.485	2.499	2.493	2.477	2.493
				(0.063)	(0.063)	(0.063)	(0.109)	(0.109)	(0.109)	(0.271)	(0.271)	(0.271)	(0.278)	(0.280)	(0.278)
			0.2	0.211	0.211	0.211	1.097	1.095	1.097	2.713	2.699	2.713	0.886	0.886	0.886
				(0.068)	(0.068)	(0.068)	(0.121)	(0.121)	(0.121)	(0.432)	(0.436)	(0.432)	(0.354)	(0.354)	(0.354)
		0.4	0	0.285	0.285	0.285	1.602	1.598	1.602	3.704	3.680	3.704	3.699	3.674	3.699
				(0.076)	(0.076)	(0.076)	(0.128)	(0.128)	(0.128)	(0.323)	(0.325)	(0.324)	(0.327)	(0.329)	(0.327)
			0.2	0.296	0.296	0.296	1.625	1.621	1.625	3.980	3.958	3.980	1.172	1.171	1.172
				(0.080)	(0.080)	(0.080)	(0.145)	(0.146)	(0.145)	(0.480)	(0.484)	(0.480)	(0.446)	(0.446)	(0.446)
	7	0	0	0.056	0.056	0.056	0.033	0.033	0.033	0.086	0.088	0.086	0.084	0.086	0.084
				(0.026)	(0.026)	(0.026)	(0.014)	(0.014)	(0.014)	(0.045)	(0.048)	(0.045)	(0.043)	(0.045)	(0.043)
			0.2	0.064	0.064	0.064	0.039	0.040	0.039	0.174	0.178	0.174	0.467	0.467	0.467
				(0.027)	(0.027)	(0.027)	(0.015)	(0.015)	(0.015)	(0.116)	(0.118)	(0.116)	(0.194)	(0.194)	(0.194)
		0.2	0	0.297	0.297	0.297	1.574	1.570	1.574	3.637	3.615	3.637	3.629	3.605	3.629
				(0.092)	(0.092)	(0.092)	(0.155)	(0.155)	(0.155)	(0.389)	(0.389)	(0.389)	(0.391)	(0.393)	(0.391)
			0.2	0.312	0.312	0.312	1.603	1.600	1.603	3.925	3.904	3.925	1.312	1.311	1.312
				(0.097)	(0.097)	(0.097)	(0.172)	(0.173)	(0.172)	(0.610)	(0.617)	(0.610)	(0.556)	(0.556)	(0.556)
		0.4	0	0.415	0.415	0.415	2.341	2.336	2.341	5.407	5.373	5.407	5.415	5.380	5.415
				(0.109)	(0.109)	(0.109)	(0.188)	(0.189)	(0.188)	(0.471)	(0.473)	(0.471)	(0.473)	(0.472)	(0.473)
			0.2	0.438	0.438	0.438	2.372	2.367	2.372	5.848	5.817	5.848	1.672	1.672	1.672
				(0.124)	(0.124)	(0.124)	(0.203)	(0.204)	(0.203)	(0.719)	(0.724)	(0.719)	(0.636)	(0.636)	(0.636)
1/45	3	0	0	0.018	0.018	0.018	0.011	0.011	0.011	0.029	0.030	0.029	0.029	0.030	0.029
				(0.005)	(0.005)	(0.005)	(0.003)	(0.003)	(0.003)	(0.010)	(0.011)	(0.010)	(0.010)	(0.010)	(0.010)
			0.2	0.020	0.020	0.020	0.013	0.013	0.013	0.058	0.060	0.058	0.153	0.153	0.153
				(0.006)	(0.006)	(0.006)	(0.004)	(0.004)	(0.004)	(0.027)	(0.028)	(0.027)	(0.045)	(0.045)	(0.045)
		0.2	0	0.091	0.091	0.091	0.477	0.476	0.477	1.104	1.098	1.104	1.099	1.093	1.099
				(0.019)	(0.019)	(0.019)	(0.033)	(0.033)	(0.033)	(0.086)	(0.086)	(0.086)	(0.085)	(0.086)	(0.085)
			0.2	0.095	0.095	0.095	0.484	0.483	0.484	1.198	1.193	1.198	0.397	0.397	0.397
				(0.021)	(0.021)	(0.021)	(0.037)	(0.037)	(0.037)	(0.132)	(0.134)	(0.132)	(0.117)	(0.117)	(0.117)
		0.4	0	0.126	0.126	0.126	0.707	0.706	0.707	1.634	1.623	1.634	1.637	1.627	1.637
				(0.024)	(0.024)	(0.024)	(0.042)	(0.043)	(0.042)	(0.107)	(0.108)	(0.107)	(0.102)	(0.103)	(0.102)
			0.2	0.133	0.133	0.133	0.718	0.716	0.718	1.769	1.760	1.769	0.532	0.532	0.532
				(0.026)	(0.026)	(0.026)	(0.047)	(0.047)	(0.047)	(0.161)	(0.163)	(0.161)	(0.138)	(0.138)	(0.138)
	5	0	0	0.039	0.039	0.039	0.023	0.024	0.023	0.061	0.063	0.061	0.060	0.062	0.060
				(0.012)	(0.012)	(0.012)	(0.007)	(0.007)	(0.007)	(0.021)	(0.022)	(0.021)	(0.021)	(0.023)	(0.021)
			0.2	0.043	0.043	0.043	0.027	0.028	0.027	0.121	0.125	0.121	0.324	0.324	0.324
				(0.013)	(0.013)	(0.013)	(0.007)	(0.008)	(0.007)	(0.057)	(0.059)	(0.057)	(0.094)	(0.094)	(0.094)
		0.2	0	0.195	0.195	0.195	1.023	1.020	1.023	2.367	2.352	2.367	2.359	2.345	2.359
				(0.043)	(0.043)	(0.043)	(0.074)	(0.075)	(0.074)	(0.189)	(0.192)	(0.189)	(0.178)	(0.181)	(0.178)
			0.2	0.201	0.201	0.201	1.034	1.032	1.034	2.565	2.553	2.565	0.872	0.872	0.872
				(0.045)	(0.045)	(0.045)	(0.078)	(0.078)	(0.078)	(0.284)	(0.286)	(0.284)	(0.253)	(0.253)	(0.253)
		0.4	0	0.275	0.275	0.275	1.526	1.523	1.526	3.528	3.505	3.528	3.519	3.497	3.518
				(0.052)	(0.052)	(0.052)	(0.090)	(0.090)	(0.090)	(0.226)	(0.228)	(0.226)	(0.219)	(0.221)	(0.219)
			0.2	0.283	0.283	0.283	1.543	1.540	1.543	3.804	3.784	3.804	1.121	1.120	1.121
				(0.054)	(0.054)	(0.054)	(0.097)	(0.097)	(0.097)	(0.346)	(0.349)	(0.346)	(0.292)	(0.292)	(0.292)
	7	0	0	0.057	0.057	0.057	0.034	0.035	0.034	0.089	0.091	0.089	0.088	0.090	0.088
				(0.017)	(0.017)	(0.017)	(0.010)	(0.010)	(0.010)	(0.031)	(0.033)	(0.031)	(0.030)	(0.033)	(0.030)
			0.2	0.062	0.062	0.062	0.041	0.041	0.041	0.177	0.182	0.177	0.476	0.476	0.476
				(0.018)	(0.018)	(0.018)	(0.011)	(0.012)	(0.011)	(0.082)	(0.084)	(0.082)	(0.137)	(0.137)	(0.137)
		0.2	0	0.285	0.285	0.285	1.496	1.493	1.496	3.462	3.441	3.462	3.466	3.446	3.466
				(0.061)	(0.061)	(0.061)	(0.107)	(0.107)	(0.107)	(0.265)	(0.266)	(0.265)	(0.271)	(0.274)	(0.271)
			0.2	0.300	0.300	0.300	1.513	1.510	1.513	3.759	3.741	3.759	1.266	1.266	1.266
				(0.067)	(0.067)	(0.067)	(0.118)	(0.119)	(0.118)	(0.413)	(0.418)	(0.413)	(0.373)	(0.373)	(0.373)
		0.4	0	0.395	0.395	0.395	2.219	2.215	2.219	5.123	5.091	5.123	5.138	5.105	5.138
				(0.076)	(0.076)	(0.076)	(0.135)	(0.135)	(0.135)	(0.335)	(0.338)	(0.335)	(0.324)	(0.325)	(0.324)
			0.2	0.412	0.412	0.412	2.253	2.249	2.253	5.532	5.501	5.532	1.644	1.644	1.644
				(0.078)	(0.078)	(0.078)	(0.146)	(0.146)	(0.146)	(0.518)	(0.524)	(0.518)	(0.420)	(0.420)	(0.420)

Note: The average ISE_1_ value is shown above each data unit, and the standard error of ISE_1_ is shown in parentheses below. *r^2^* represents the measure of linkage disequilibrium, *r^2^* equals to 0 means there is linkage equilibrium between SNP.

**Table 4:** The means and standard errors (in the parenthesis) of PMSE for association analysis of simulated quantitative trait based on SFDAT, OLS and Smooth and on linkage equilibrium and linkage disequilibrium.

Associated Variants Proportion	c	Negative Effect Proportion	*r* ^2^	Common Variants			Low-Frequency Variants			Rare Variants			Mix Variants		
				SFDAT	OLS	Smooth	SFDAT	OLS	Smooth	SFDAT	OLS	Smooth	SFDAT	OLS	Smooth
1/180	3	0	0	0.260	0.260	0.260	0.230	0.226	0.225	0.097	0.100	0.097	0.097	0.100	0.097
				(0.108)	(0.107)	(0.107)	(0.068)	(0.068)	(0.068)	(0.045)	(0.044)	(0.045)	(0.046)	(0.046)	(0.046)
			0.2	0.173	0.174	0.174	0.197	0.198	0.197	0.083	0.086	0.083	0.170	0.170	0.170
				(0.039)	(0.039)	(0.039)	(0.049)	(0.049)	(0.049)	(0.037)	(0.037)	(0.037)	(0.045)	(0.045)	(0.045)
		0.2	0	0.247	0.247	0.247	0.220	0.223	0.222	0.097	0.099	0.097	0.095	0.097	0.095
				(0.101)	(0.101)	(0.101)	(0.061)	(0.061)	(0.061)	(0.045)	(0.045)	(0.045)	(0.044)	(0.044)	(0.044)
			0.2	0.176	0.177	0.177	0.198	0.200	0.198	0.083	0.086	0.083	0.168	0.168	0.168
				(0.040)	(0.040)	(0.040)	(0.053)	(0.053)	(0.053)	(0.039)	(0.039)	(0.039)	(0.045)	(0.045)	(0.045)
		0.4	0	0.250	0.251	0.250	0.220	0.221	0.220	0.097	0.100	0.097	0.097	0.100	0.097
				(0.111)	(0.111)	(0.110)	(0.062)	(0.062)	(0.062)	(0.046)	(0.046)	(0.046)	(0.046)	(0.046)	(0.046)
			0.2	0.176	0.176	0.176	0.200	0.202	0.200	0.084	0.087	0.084	0.170	0.170	0.170
				(0.040)	(0.040)	(0.040)	(0.054)	(0.054)	(0.054)	(0.040)	(0.041)	(0.040)	(0.047)	(0.047)	(0.047)
	5	0	0	0.523	0.523	0.523	0.460	0.460	0.459	0.196	0.198	0.196	0.193	0.196	0.193
				(0.226)	(0.226)	(0.226)	(0.134)	(0.134)	(0.134)	(0.099)	(0.099)	(0.099)	(0.097)	(0.096)	(0.097)
			0.2	0.362	0.362	0.362	0.413	0.414	0.413	0.171	0.174	0.171	0.339	0.340	0.339
				(0.084)	(0.084)	(0.084)	(0.105)	(0.106)	(0.105)	(0.084)	(0.084)	(0.084)	(0.094)	(0.094)	(0.094)
		0.2	0	0.536	0.537	0.536	0.470	0.468	0.467	0.199	0.202	0.199	0.198	0.201	0.199
				(0.239)	(0.239)	(0.239)	(0.136)	(0.136)	(0.136)	(0.103)	(0.103)	(0.103)	(0.096)	(0.096)	(0.096)
			0.2	0.364	0.365	0.365	0.407	0.409	0.407	0.166	0.170	0.166	0.345	0.346	0.345
				(0.085)	(0.085)	(0.085)	(0.104)	(0.104)	(0.104)	(0.085)	(0.085)	(0.085)	(0.094)	(0.094)	(0.094)
		0.4	0	0.520	0.521	0.520	0.470	0.469	0.468	0.202	0.205	0.202	0.197	0.200	0.197
				(0.228)	(0.228)	(0.228)	(0.141)	(0.141)	(0.141)	(0.105)	(0.104)	(0.105)	(0.095)	(0.095)	(0.095)
			0.2	0.363	0.364	0.364	0.407	0.409	0.407	0.167	0.170	0.167	0.343	0.344	0.343
				(0.084)	(0.084)	(0.083)	(0.101)	(0.101)	(0.101)	(0.083)	(0.083)	(0.083)	(0.094)	(0.094)	(0.094)
	7	0	0	0.754	0.754	0.754	0.670	0.668	0.666	0.281	0.284	0.281	0.280	0.282	0.280
				(0.331)	(0.331)	(0.331)	(0.200)	(0.200)	(0.200)	(0.146)	(0.145)	(0.146)	(0.143)	(0.142)	(0.143)
			0.2	0.521	0.522	0.521	0.590	0.592	0.590	0.239	0.242	0.239	0.498	0.499	0.498
				(0.120)	(0.120)	(0.120)	(0.150)	(0.150)	(0.150)	(0.117)	(0.117)	(0.117)	(0.138)	(0.138)	(0.138)
		0.2	0	0.762	0.763	0.762	0.670	0.670	0.668	0.283	0.286	0.283	0.279	0.282	0.279
				(0.315)	(0.315)	(0.315)	(0.190)	(0.190)	(0.190)	(0.141)	(0.140)	(0.141)	(0.144)	(0.143)	(0.144)
			0.2	0.528	0.528	0.528	0.595	0.597	0.595	0.243	0.247	0.243	0.502	0.503	0.502
				(0.123)	(0.123)	(0.123)	(0.150)	(0.150)	(0.150)	(0.126)	(0.126)	(0.126)	(0.136)	(0.136)	(0.136)
		0.4	0	0.751	0.752	0.751	0.660	0.662	0.660	0.273	0.276	0.273	0.277	0.279	0.277
				(0.323)	(0.323)	(0.323)	(0.187)	(0.187)	(0.187)	(0.142)	(0.141)	(0.141)	(0.143)	(0.143)	(0.143)
			0.2	0.524	0.524	0.524	0.597	0.599	0.597	0.239	0.242	0.239	0.509	0.510	0.509
				(0.125)	(0.126)	(0.125)	(0.153)	(0.154)	(0.153)	(0.116)	(0.117)	(0.116)	(0.138)	(0.138)	(0.138)
1/90	3	0	0	0.487	0.488	0.487	0.430	0.431	0.430	0.184	0.186	0.184	0.181	0.183	0.181
				(0.153)	(0.153)	(0.153)	(0.099)	(0.099)	(0.099)	(0.067)	(0.067)	(0.067)	(0.068)	(0.067)	(0.068)
			0.2	0.335	0.335	0.335	0.379	0.380	0.379	0.156	0.159	0.156	0.323	0.324	0.323
				(0.066)	(0.066)	(0.066)	(0.087)	(0.087)	(0.087)	(0.062)	(0.062)	(0.062)	(0.073)	(0.073)	(0.073)
		0.2	0	0.486	0.486	0.486	0.430	0.430	0.429	0.183	0.186	0.184	0.182	0.184	0.182
				(0.156)	(0.156)	(0.156)	(0.097)	(0.097)	(0.097)	(0.068)	(0.068)	(0.068)	(0.066)	(0.066)	(0.066)
			0.2	0.338	0.339	0.339	0.388	0.389	0.388	0.157	0.160	0.157	0.324	0.324	0.324
				(0.066)	(0.066)	(0.066)	(0.087)	(0.087)	(0.087)	(0.063)	(0.063)	(0.063)	(0.072)	(0.072)	(0.072)
		0.4	0	0.491	0.491	0.491	0.430	0.431	0.430	0.184	0.186	0.184	0.183	0.185	0.183
				(0.160)	(0.160)	(0.160)	(0.098)	(0.098)	(0.098)	(0.068)	(0.068)	(0.068)	(0.067)	(0.067)	(0.067)
			0.2	0.342	0.342	0.342	0.383	0.385	0.383	0.159	0.162	0.159	0.324	0.325	0.324
				(0.067)	(0.067)	(0.067)	(0.085)	(0.085)	(0.085)	(0.064)	(0.064)	(0.064)	(0.073)	(0.073)	(0.073)
	5	0	0	1.024	1.025	1.025	0.920	0.916	0.915	0.383	0.386	0.383	0.375	0.378	0.375
				(0.333)	(0.333)	(0.333)	(0.218)	(0.218)	(0.218)	(0.141)	(0.141)	(0.141)	(0.140)	(0.140)	(0.140)
			0.2	0.711	0.711	0.711	0.796	0.798	0.796	0.316	0.320	0.316	0.681	0.682	0.681
				(0.141)	(0.141)	(0.141)	(0.179)	(0.179)	(0.179)	(0.125)	(0.125)	(0.125)	(0.162)	(0.162)	(0.162)
		0.2	0	1.043	1.044	1.043	0.910	0.909	0.908	0.380	0.382	0.380	0.380	0.382	0.380
				(0.338)	(0.338)	(0.338)	(0.211)	(0.210)	(0.211)	(0.145)	(0.145)	(0.145)	(0.139)	(0.138)	(0.139)
			0.2	0.712	0.713	0.712	0.813	0.815	0.813	0.319	0.323	0.319	0.689	0.689	0.689
				(0.141)	(0.142)	(0.142)	(0.184)	(0.184)	(0.184)	(0.125)	(0.126)	(0.125)	(0.153)	(0.153)	(0.153)
		0.4	0	1.029	1.030	1.029	0.910	0.907	0.905	0.382	0.385	0.382	0.377	0.380	0.377
				(0.338)	(0.338)	(0.338)	(0.215)	(0.215)	(0.215)	(0.139)	(0.139)	(0.139)	(0.143)	(0.143)	(0.143)
			0.2	0.712	0.712	0.712	0.809	0.811	0.809	0.330	0.334	0.330	0.686	0.687	0.687
				(0.136)	(0.136)	(0.136)	(0.185)	(0.185)	(0.185)	(0.131)	(0.132)	(0.131)	(0.159)	(0.159)	(0.159)
	7	0	0	1.474	1.475	1.474	1.310	1.314	1.312	0.546	0.549	0.546	0.547	0.550	0.547
				(0.487)	(0.487)	(0.487)	(0.305)	(0.304)	(0.305)	(0.202)	(0.202)	(0.202)	(0.202)	(0.202)	(0.202)
			0.2	1.041	1.042	1.041	1.155	1.158	1.155	0.465	0.470	0.465	1.002	1.003	1.002
				(0.209)	(0.209)	(0.209)	(0.259)	(0.259)	(0.259)	(0.185)	(0.185)	(0.185)	(0.224)	(0.225)	(0.224)
		0.2	0	1.504	1.505	1.504	1.320	1.320	1.318	0.552	0.555	0.552	0.553	0.556	0.553
				(0.492)	(0.492)	(0.492)	(0.312)	(0.312)	(0.312)	(0.221)	(0.221)	(0.221)	(0.210)	(0.209)	(0.210)
			0.2	1.035	1.036	1.035	1.191	1.193	1.191	0.459	0.463	0.459	0.998	0.999	0.998
				(0.205)	(0.205)	(0.205)	(0.274)	(0.274)	(0.274)	(0.185)	(0.184)	(0.185)	(0.229)	(0.229)	(0.229)
		0.4	0	1.497	1.497	1.497	1.330	1.332	1.330	0.550	0.553	0.550	0.550	0.552	0.550
				(0.484)	(0.484)	(0.484)	(0.310)	(0.310)	(0.310)	(0.202)	(0.202)	(0.202)	(0.201)	(0.199)	(0.201)
			0.2	1.047	1.048	1.047	1.189	1.191	1.189	0.471	0.476	0.471	1.003	1.004	1.003
				(0.207)	(0.207)	(0.207)	(0.266)	(0.266)	(0.266)	(0.195)	(0.196)	(0.195)	(0.228)	(0.228)	(0.227)
1/45	3	0	0	0.949	0.950	0.949	0.840	0.844	0.842	0.354	0.357	0.354	0.350	0.353	0.350
				(0.236)	(0.236)	(0.236)	(0.161)	(0.162)	(0.161)	(0.099)	(0.099)	(0.099)	(0.098)	(0.098)	(0.098)
			0.2	0.658	0.658	0.658	0.745	0.748	0.745	0.299	0.303	0.299	0.636	0.637	0.636
				(0.115)	(0.115)	(0.115)	(0.153)	(0.153)	(0.153)	(0.097)	(0.097)	(0.097)	(0.126)	(0.126)	(0.126)
		0.2	0	0.960	0.960	0.960	0.840	0.837	0.836	0.351	0.354	0.351	0.357	0.360	0.357
				(0.239)	(0.239)	(0.239)	(0.158)	(0.158)	(0.158)	(0.096)	(0.096)	(0.096)	(0.100)	(0.100)	(0.100)
			0.2	0.667	0.667	0.667	0.754	0.756	0.754	0.306	0.310	0.306	0.633	0.634	0.633
				(0.116)	(0.116)	(0.116)	(0.156)	(0.156)	(0.156)	(0.097)	(0.098)	(0.097)	(0.127)	(0.127)	(0.127)
		0.4	0	0.964	0.964	0.964	0.850	0.847	0.846	0.355	0.357	0.355	0.356	0.359	0.356
				(0.244)	(0.244)	(0.244)	(0.158)	(0.158)	(0.158)	(0.100)	(0.099)	(0.100)	(0.096)	(0.095)	(0.096)
			0.2	0.669	0.669	0.669	0.752	0.754	0.752	0.305	0.309	0.305	0.637	0.637	0.637
				(0.113)	(0.113)	(0.113)	(0.156)	(0.156)	(0.156)	(0.106)	(0.108)	(0.106)	(0.122)	(0.122)	(0.122)
	5	0	0	2.019	2.020	2.019	1.770	1.768	1.765	0.738	0.740	0.738	0.743	0.747	0.743
				(0.498)	(0.498)	(0.498)	(0.332)	(0.333)	(0.332)	(0.211)	(0.211)	(0.211)	(0.204)	(0.204)	(0.204)
			0.2	1.399	1.399	1.399	1.573	1.576	1.573	0.630	0.635	0.630	1.336	1.337	1.336
				(0.247)	(0.247)	(0.247)	(0.342)	(0.342)	(0.342)	(0.215)	(0.216)	(0.215)	(0.273)	(0.273)	(0.273)
		0.2	0	2.050	2.050	2.050	1.800	1.799	1.796	0.760	0.763	0.760	0.732	0.735	0.732
				(0.529)	(0.529)	(0.529)	(0.341)	(0.341)	(0.341)	(0.212)	(0.211)	(0.212)	(0.211)	(0.210)	(0.211)
			0.2	1.413	1.414	1.413	1.611	1.615	1.611	0.634	0.639	0.634	1.353	1.354	1.353
				(0.256)	(0.256)	(0.256)	(0.342)	(0.343)	(0.342)	(0.213)	(0.213)	(0.213)	(0.271)	(0.271)	(0.271)
		0.4	0	2.051	2.052	2.051	1.810	1.813	1.811	0.752	0.756	0.752	0.755	0.758	0.755
				(0.508)	(0.508)	(0.508)	(0.353)	(0.353)	(0.353)	(0.220)	(0.219)	(0.220)	(0.214)	(0.213)	(0.214)
			0.2	1.400	1.400	1.400	1.626	1.629	1.626	0.633	0.638	0.633	1.357	1.358	1.357
				(0.244)	(0.244)	(0.244)	(0.350)	(0.350)	(0.350)	(0.213)	(0.214)	(0.213)	(0.272)	(0.272)	(0.272)
	7	0	0	2.944	2.944	2.944	2.600	2.599	2.596	1.079	1.084	1.079	1.071	1.074	1.071
				(0.735)	(0.735)	(0.735)	(0.491)	(0.490)	(0.491)	(0.300)	(0.299)	(0.300)	(0.299)	(0.297)	(0.299)
			0.2	2.034	2.035	2.034	2.276	2.281	2.276	0.916	0.923	0.916	1.963	1.964	1.963
				(0.352)	(0.352)	(0.352)	(0.488)	(0.489)	(0.488)	(0.317)	(0.319)	(0.317)	(0.414)	(0.414)	(0.414)
		0.2	0	2.987	2.988	2.987	2.630	2.633	2.630	1.099	1.102	1.099	1.078	1.081	1.078
				(0.738)	(0.739)	(0.738)	(0.501)	(0.501)	(0.501)	(0.319)	(0.318)	(0.319)	(0.306)	(0.304)	(0.306)
			0.2	2.049	2.050	2.049	2.333	2.338	2.333	0.922	0.928	0.922	1.980	1.982	1.980
				(0.362)	(0.362)	(0.362)	(0.490)	(0.490)	(0.490)	(0.308)	(0.308)	(0.308)	(0.402)	(0.402)	(0.402)
		0.4	0	2.954	2.955	2.954	2.640	2.643	2.640	1.093	1.096	1.093	1.107	1.110	1.107
				(0.749)	(0.749)	(0.749)	(0.500)	(0.500)	(0.500)	(0.320)	(0.320)	(0.320)	(0.320)	(0.318)	(0.320)
			0.2	2.053	2.054	2.053	2.365	2.369	2.365	0.928	0.936	0.928	1.987	1.988	1.987
				(0.356)	(0.355)	(0.356)	(0.511)	(0.511)	(0.511)	(0.315)	(0.316)	(0.315)	(0.420)	(0.420)	(0.420)

Note: The average PMSE value is shown above each data unit, and the standard deviation of PMSE is shown in parentheses below. *r^2^* represents the measure of linkage disequilibrium, *r^2^* equals to 0 means there is linkage equilibrium between SNP.

**Table 5:** Type I error rates of association analysis of simulated quantitative trait of SFDAT, OLS and Smooth based on 1000 simulated replicates for linkage equilibrium and linkage equilibrium.

Sample Size	Significant Level α	Linkage Equilibrium			Linkage Disequilibrium		
		SFDAT	OLS	Smooth	SFDAT	OLS	Smooth
500	0.05	0.0457	0.0492	0.0457	0.0420	0.0468	0.0420
	0.01	0.0088	0.0098	0.0088	0.0085	0.0095	0.0085
	0.001	0.0006	0.0006	0.0006	0.0005	0.0005	0.0005
	0.0001	0.0002	0.0002	0.0002	0.0000	0.0000	0.0000
1000	0.05	0.0481	0.0524	0.0487	0.0476	0.0524	0.0484
	0.01	0.0088	0.0100	0.0089	0.0093	0.0113	0.0094
	0.001	0.0009	0.0012	0.0009	0.0011	0.0012	0.0011
	0.0001	0.0001	0.0001	0.0001	0.0001	0.0001	0.0001
1500	0.05	0.0453	0.0502	0.0471	0.0421	0.0478	0.0439
	0.01	0.0115	0.0126	0.0118	0.0089	0.0106	0.0091
	0.001	0.0018	0.0020	0.0018	0.0008	0.0012	0.0008
	0.0001	0.0002	0.0002	0.0002	0.0000	0.0000	0.0000
2000	0.05	0.0403	0.0477	0.0442	0.0435	0.0479	0.0435
	0.01	0.0085	0.0101	0.0090	0.0072	0.0080	0.0072
	0.001	0.0009	0.0011	0.0010	0.0008	0.0009	0.0008
	0.0001	0.0001	0.0001	0.0001	0.0000	0.0000	0.0000
2500	0.05	0.0364	0.0486	0.0449	0.0366	0.0506	0.0475
	0.01	0.0079	0.0112	0.0101	0.0074	0.0100	0.0089
	0.001	0.0014	0.0014	0.0014	0.0007	0.0009	0.0007
	0.0001	0.0003	0.0004	0.0004	0.0000	0.0000	0.0000

**Table 6:** The number of SNPs and the *p*-value of the association analysis of each gene region of Culm habit based on SFDAT.

Chromosome	Gene Region	No. of SNPs in Test	*p*-Value	Chromosome	Gene Region	No. of SNPs in Test	*p*-Value
1	1	1000	0.8764	4	13	1422	0.0619
1	2	1000	0.9856	5	14	1000	0.0119
1	3	1000	0.0326	5	15	1569	0.0505
1	4	1000	0.0484	6	16	1000	0.1630
1	5	1706	0.0798	6	17	1846	0.3338
2	6	1000	0.1591	7	18	1792	0.3248
2	7	1000	0.4186	8	19	1957	0.1973
2	8	1500	0.3048	9	20	1682	0.1331
3	9	1000	0.0389	10	21	1484	0.2575
3	10	1000	0.5562	11	22	1000	0.8152
3	11	1963	0.9100	11	23	1453	0.3943
4	12	1000	0.9029	12	24	1811	0.0936

Note: Causal SNP *D10* and *D14* of the Culm habit are located in the 4th and 9th gene region, respectively.

**Table 7:** The gene regions of candidate SNP identified by SFDAT, OLS and Smooth at different significant levels.

Traits	Florets Per Panicle			Brown Rice Seed Length			Flowering Time at Aberdeen		
Significant Level α	SSD1			GS3			Hd1		
	SFDAT	OLS	Smooth	SFDAT	OLS	Smooth	SFDAT	OLS	Smooth
0.05	4,7	1,2,3,4,5,7,8	1,2,3,4,5,7,8	4,6,8	1,2,3,4,5,6,7,8	1,2,3,4,5,6,7,8	3,4,5	3,4,5,7	3,4,5
0.01	4,7	1,3,4,5,7,8	1,3,4,5,7,8	6,8	1,2,3,4,5,6,7,8	1,2,3,4,5,6,7,8	3,4	3,4,5	3,4,5
0.001	4	1,3,4,7,8	1,3,4,7,8	6,8	1,2,3,4,5,6,7,8	1,2,3,4,5,6,7,8	3	3,4,5	3

Note: *SSD1* is the candidate SNP of Florets per panicle which locate in gene region 4; *GS3* is the candidate SNP of Brown rice seed length which locate in gene region 6; *Hd1* is the candidate SNP of Flowering time at Aberdeen which were located in gene region 3.

## Data Availability

Publicly available datasets were analyzed in this study. The data can be found here: www.ricediversity.org/44kgwas (accessed on 1 December 2022) and www.gramene.org (accessed on 1 December 2022).
